# Silver nanoparticle hydrogen peroxide composite mitigates resistant *Escherichia coli* dissemination driven by poultry waste biosecurity failures

**DOI:** 10.1038/s41598-026-61245-8

**Published:** 2026-07-16

**Authors:** Basma Badawy, Mohamed Z. Sayed-Ahmed, Mona M. Elsayed, Mohammed Al-Rasheed, Wael El-Deeb, Mohamed Sabry Ahmed, Abdulrhman K. Alhaider, Mahmoud H. A. Mohamed, Mai F. Saad, Manal A. Al-Ashery, Yara F. H. El-Basrey, Mohamed Abdo Rizk

**Affiliations:** 1 Department of Hygiene and Zoonoses, Faculty of Veterinary Medicine, Mansoura University, Mansoura, 35516 Egypt; 2 Department of Clinical Practice, Pharmacy Practice Research Unit, College of Pharmacy, Jazan University, Jazan, 45142 Saudi Arabia; 3 Department of Clinical Sciences, College of Veterinary Medicine, King Faisal University, P.O. Box 400, Al-Ahsa, 31982 Saudi Arabia; 4 Department of Veterinary Public Health, Faculty of Veterinary Medicine, Zagazig University, Zagazig, 44511 Egypt; 5 Avian and Rabbit Medicine Department, Faculty of Veterinary Medicine, Zagazig University, Zagazig, 44511 Egypt

**Keywords:** Extensively drug-resistant *Escherichia coli*, Silver nanoparticle composite, Environmental biosecurity, MAR index, Biotechnology, Environmental sciences, Microbiology

## Abstract

This two-phase study evaluated biosecurity vulnerabilities across 100 commercial poultry farms via a field survey and an in vitro assessment of hydrogen peroxide (H_2_O_2_) versus a silver nanoparticle–hydrogen peroxide (AgNPs– H_2_O_2_) composite. Survey metrics revealed critical baseline deficiencies: 80% of farmers lacked antimicrobial resistance (AMR) awareness, 90% practiced no litter treatment, and 70% of untreated waste was sold directly to aquaculture. Molecular analysis of 192 litter samples verified a 72.4% *E. coli* prevalence, with Multiple Antibiotic Resistance (MAR) indices peaking at 0.90. Phenotypic profiling showed high resistance to ampicillin (89.9%) and amoxicillin-clavulanic acid (61.2%), whereas colistin demonstrated 100% susceptibility. A significant co-resistance was identified between imipenem and tetracycline (φ = 0.65, p_adj < 0.001) and between cefotaxime and ceftazidime (φ = 0.58, p_adj < 0.001), *P* < 0.01) after Benjamini-Hochberg FDR correction. Concurrently, the AgNPs– H_2_O_2_ composite exhibited superior efficacy with a Minimum Inhibitory Concentration (MIC) of 3.125 µg/mL, proving four-fold more potent than standalone H_2_O_2_. Time-kill kinetics demonstrated complete bacterial reduction within 24 h (*P* < 0.001), successfully suppressing the post-6-hour regrowth observed with H_2_O_2_ alone. While this study is limited by its regional geographical scope and the lack of molecular characterization for specific resistance genes, it conclusively identifies veterinary supervision deficits (*P* < 0.0001) as a driver of extensively drug-resistant (XDR) transmission. Ultimately, the AgNPs– H_2_O_2_ composite offers a promising in vitro One Health biosecurity strategy, achieving complete bacterial reduction under controlled conditions at a 75% lower concentration than standalone H_2_O_2_, pending field validation.

**Supplementary Information:**

The online version contains supplementary material available at 10.1038/s41598-026-61245-8.

## Introduction

The poultry industry is a cornerstone of global food security and economic stability, especially in Egypt. However, bacterial infections, particularly *E. coli*, pose a serious threat to intensive production and serve as a critical indicator of environmental hygiene. The emergence of Multidrug-Resistant (MDR) and Extensively Drug-Resistant (XDR) strains, fueled by excessive antibiotic use, has further complicated disease management^1^. Animal gut bacteria develop resistance to the antimicrobial agents that have been utilized when they are exposed to sub-therapeutic concentrations and frequencies of these agents. Chicken as food animals, where antibiotics are frequently given to whole flocks as growth boosters, and *E. coli* as a crucial component of the endogenous microflora that quickly becomes resistant to antibiotics that birds eat. Furthermore, it is well known that *E. coli* has the ability to transmit antibiotic resistance genes to other strains and other bacteria^2^.

MDR *E. coli* in poultry has serious zoonotic and public health hazard that can spread to humans and other One Health components. Determinants of antimicrobial resistance (AMR) can also be transferred to other bacterial pathogens that affect animal and human via transferable genetic materials^3^, Gumede et al. 2026). The rising emergence of MDR and XDR strains, driven by excessive antibiotic use, has further complicated disease management. Research indicates that conventional farming systems harbor significantly higher resistance levels compared to organic ones^4^. This problem is exacerbated by the physiological excretion of unmetabolized antibiotics into the litter, creating “hotspots” for horizontal gene transfer and the spread of critical resistance markers^5,6^. Furthermore, the documented zoonotic spread of resistant genotypes among poultry and humans highlights a profound “One Health” alarm^7^.

Poultry litter acts as a major reservoir for many enteric pathogens, where environmental management directly affects bacterial persistence and spread^8^. High prevalence rates of *E. coli* in broiler houses are often linked to inadequate biosecurity measures as reported in some recent surveillance across the Nile Delta^9,10^. Various food-borne disease can result from failure to combat these pathogens in poultry litter. The emergence and spreading of MDR bacteria in both veterinary and human medicine are strongly associated with antibiotic use in pathogenic bacteria and the endogenous bacterial flora of exposed animals and humans. This widespread resistance development is largely driven by active field practices, where some poultry growers still administer antibiotics as growth promoters or for routine prophylactic disease prevention. Consequently, it is highly probable that un-metabolized antibiotic residues are excreted and accumulated within the litter matrix, potentially exposing the environmental microflora to continuous selective pressure and subsequently increasing the risk of emerging resistance^11^. Nevertheless, it should be noted that direct chemical quantification of these residues was beyond the scope of the present study, and this mechanism remains an inferred correlation based on the reported farm practices.

The systemic “knowledge-practice gap” is a significant challenge in waste management^12^. In developing poultry sectors, the direct disposal of untreated poultry manure into agricultural fields and aquaculture is a common and hazardous practice, leading to severe environmental deterioration and public health risks. Poultry litter has traditionally been applied to increase primary production and create natural food webs in aquaculture ponds, therefore there is a strong economic incentive for this approach despite these hazards^13^. Using untreated litter allows XDR bacteria to directly enter the human food chain, while it promotes the early development of stocked juveniles. A shift to a circular bioeconomy is necessary to eliminate this danger. Cutting-edge techniques, such as making Microbial Protein Feed (MPF) from fermented agricultural waste, provide a sustainable way to lower carbon emissions while still producing high-quality protein^14^. However, the complete reduction of resistant microorganisms is necessary for such recycling procedures to be safe.

The failure of traditional biosecurity against XDR pathogens in poultry waste necessitates innovative antimicrobial strategies, with nanotechnology offering a promising frontier. Nanoparticles (NPs), including metallic NPs and composite formulations, have demonstrated potent multi-target antimicrobial activity, the capacity to overcome existing resistance mechanisms, and synergistic effects when combined with oxidizing agents^15,16^. The enhanced antimicrobial mechanism of the AgNPs– H_2_O_2_ nanocomposite is characterized by a cooperative pathway that overcomes the limitations of individual agents. Firstly, AgNPs serve as a protective carrier and a stabilizing platform that prevents the premature decomposition of H_2_O_2_, ensuring its regulated release and sustained potency against resilient pathogens^17^. Recent evidence further suggests that H_2_O_2_ acts as a central core in the AgNP signaling pathway, where H_2_O_2_ increases cell membrane permeability, thereby facilitating the internal localization of silver ions and nanoparticles^18^. Once inside, AgNPs act as effective catalysts that accelerate the decomposition of H_2_O_2_ into highly reactive hydroxyl radicals (OH-), creating an intensified ‘oxidative burst’^19^. This localized generation of reactive oxygen species (ROS), combined with the silver-mediated disruption of metabolic enzymes and DNA fragmentation, leads to a significant reduction in the Minimum Inhibitory Concentration (MIC) and rapid pathogen reduction.

Despite the global concern regarding antimicrobial resistance (AMR), a significant research gap persists in integrating field-level management factors with the environmental dissemination of extensively drug-resistant (XDR) pathogens. Specifically, there is a critical lack of data correlating the absence of professional veterinary supervision and informal waste disposal practices with the actual prevalence of XDR *E. coli* in poultry environments. While many studies focus solely on microbiological detection, the ‘supervision gap’ in biosecurity decisions remains largely unquantified. Furthermore, there is an urgent need for sustainable, nanotechnology-based interventions that can overcome the limitations of traditional chemical disinfectants. This study addresses these unresolved issues by: (1) employing a structured questionnaire to link management loopholes and the lack of expert oversight directly to microbiological findings, and (2) evaluating combined efficacy and potential of an AgNPs–H_**2**_O_**2**_ nanocomposite as a highly effective, eco-friendly alternative for the control and mitigation of environmental XDR strain. By addressing the documented lack of effective waste treatment at the farm level, this study combined formulation as a robust One Health solution for mitigating the environmental spread of XDR E. coli. Therefore, this study aimed to assess biosecurity practices and antimicrobial resistance dynamics in Egyptian broiler farms, and to explore potential nanotechnology-based disinfection strategies. Specifically, the objectives were to: (i) assess current biosecurity practices and waste management protocols in Egyptian broiler farms using a structured questionnaire, with emphasis on the role of professional veterinary supervision; (ii) investigate the prevalence and antimicrobial resistance profiles of Escherichia coli isolated from poultry litter, with particular focus on extensively drug-resistant (XDR) strains of public health concern; (iii) evaluate the association between identified biosecurity gaps and the occurrence of XDR *E. coli* within a One Health framework; (iv) determine the in vitro bactericidal efficacy of the combined AgNPs–H₂O₂ nanocomposite against these environmental XDR strains as a potential sustainable disinfection approach.

## Materials and methods

### Study area, timeline, and experimental design

This study was conducted using a two-phase approach to evaluate biosecurity gaps and the prevalence of XDR *E. coli* in poultry waste management.

**Phase I: Field survey and waste management assessment (N = 100 farms)**: A comprehensive cross-sectional survey was conducted across 100 commercial broiler farms in the Dakahlia Governorate, Egypt, from March 2023 to June 2024. The survey aimed to assess current litter disposal practices, biosecurity protocols, and farmers’ awareness of AMR. Data were collected through structured face-to-face interviews with farm managers using a validated questionnaire. Before conducting the interviews, we obtained informed oral consent from each participant. To protect the privacy of the farm owners, all collected data were strictly anonymized before analysis (Appendix S1).

**Phase II: Microbiological analysis (N = 8 representative farms)**: To evaluate the microbial burden and resistance profiles, a representative subset of 8 farms was strategically selected for intensive sampling. Selection criteria for these farms included geographic distribution across the governorate and representative waste management practices identified in Phase I. From these 8 farms, a total of 192 fresh litter samples (24 samples per farm) were collected immediately post-depopulation. Litter samples were collected using a standardized composite sampling technique. Briefly, sub-samples were obtained from 15 to 20 different locations per house following a zig-zag pattern, including areas beneath waterers and feeders. Samples were taken from a depth of 5–10 cm after removing the uppermost surface layer. These sub-samples were thoroughly pooled in sterile polyethylene bags to form a representative composite sample for each farm, then transported at 4 °C for immediate microbiological processing.

### Sampling strategy and sample size justification

 To evaluate the microbial burden and resistance profiles, a subset of 8 farms was selected from the 100-farm Phase I survey using a criterion-based convenience sampling approach. Farm enrollment was governed by four pre-specified eligibility criteria derived from the Phase I field survey dataset: (i) geographic distribution across distinct sub-districts of Dakahlia Governorate to represent spatial heterogeneity; (ii) a documented history of intensive antibiotic usage as identified during Phase I interviews; (iii) confirmed commercial disposal of poultry litter to aquaculture operations, reflecting the primary One Health biosecurity gap under investigation; and (iv) production scale within the 5,000–10,000 bird range with deep wood-shaving litter under intensive floor-rearing management.

The 8 enrolled farms represent all farms from the 100 farms surveyed in Phase I that simultaneously satisfied all four eligibility criteria and provided full, unconditional cooperation for intensive post-depopulation sampling. Random sampling from the full cohort was not operationally feasible, as the majority of private farm owners in the Egyptian poultry sector declined access for scientific sampling — a well-documented constraint in unregulated agricultural systems where farm visits are not governmentally mandated. Enrollment was therefore contingent on both criterion eligibility and voluntary participation, consistent with a criterion-based convenience sampling design. The adequacy of the resultant sample size was verified statistically at the level of individual litter samples rather than farms. A total of 192 samples were collected (24 per farm). Using the standard formula for prevalence estimation — n = Z² × p(1 − p) / d² — with Z = 1.96 (95% confidence level), an expected *E. coli* prevalence of *p* = 0.70 (based on regional poultry litter data from the Eastern Nile Delta), and a desired precision of d = ± 6.5%, the minimum required sample size was calculated as *n* = 191. The actual sample size of 192 therefore meets this threshold, providing sufficient statistical precision to estimate *E. coli* prevalence and resistance rates at the sample level. It is explicitly acknowledged that the number of farms (*n* = 8) was determined by access feasibility and criterion eligibility rather than formal farm-level power analysis, and that farm-level inferences should be interpreted within this constraint. Sampling was strategically performed immediately post-depopulation to ensure the collection of fresh litter and to ensure bacterial viability and representative flock’s microbiota. Immediate sampling prevents the loss of bacterial viability due to litter dryness or environmental variations and ensures that the isolates that recovered are representative of the flock’s microbiota before start of any terminal disinfection processes. All examined farms exhibited a mismanagement of poultry litter either its improper disposal in open environments or unregulated resale as aquaculture feed which constitutes a primary driver for the environmental dissemination of AMR. All collected samples were transferred under aseptic conditions to Hygiene and Zoonoses laboratory, Faculty of Veterinary Medicine, Mansoura University for bacteriological analysis.

### Ethical considerations and biosafety

All methods were carried out in accordance with relevant guidelines and regulations. The study protocol, encompassing the anonymous field survey and environmental microbiological analysis, was formally reviewed and approved by the Institutional Research Ethics Committee of the Faculty of Veterinary Medicine, Mansoura University (Approval Code: MU-IACUC (VMR.26.04.1. R1). For the field survey (Phase I), informed oral consent was obtained from all participants, and the study was conducted in accordance with the ethical principles of the Declaration of Helsinki. The institutional ethics committee specifically approved the use of oral consent and waived the requirement for written informed consent, as the survey was strictly anonymous, posed no risk to participants, and did not collect any personal identifiers. For the microbiological analysis (Phase II), poultry litter samples were collected as environmental waste material post-depopulation. All laboratory procedures were conducted under Biosafety Level 2 (BSL-2) conditions at the Faculty of Veterinary Medicine, Mansoura University. Bacterial isolation and nanoparticle-disinfectant testing were performed within a Class II Biosafety Cabinet to prevent aerosol exposure. Standard Personal Protective Equipment (PPE) was strictly utilized. To ensure environmental safety, all contaminated materials and multidrug-resistant cultures were sterilized via autoclaving at 121 °C for 20 min prior to disposal.

### Bacterial isolation and identification

#### Bacterial isolation and biochemical identification

All collected litter samples (25 g or 25 ml) were aseptically placed into sterile Difco-buffered peptone water (BPW) (Oxoid, UK) then incubated at 37 °C for 24 h. Then, loopful of inoculated broths were subcultured on eosin methylene blue (EMB) agar (Oxoid) and incubated at 37 °C for 24 h. Suspected colonies were picked and subjected to standard biochemical methods, urea hydrolysis, H2S production on triple sugar iron agar, lysine decarboxylation, indole, methyl red test, Voges-Proskauer test and citrate utilization test as method described by Botros et al.^9^.

#### Molecular confirmation of E. coli isolates

Genomic DNA was extracted using the thermal lysis (boiling) technique as performed previously by^20^. Molecular identification targeted the 16 S rRNA gene using primers 27 F (5′-AGAGTTTGATCCTGGCTCAG-3′) and 1492R (5′-GGTTACCTTGTTACGACTT-3′)^21^. The PCR reaction (25 µL final volume) consisted of 2× PCR Master Mix, forward and reverse primers, and the DNA template. Thermal cycling conditions included an initial denaturation at 94 °C for 5 min, followed by 30 cycles of denaturation (94 °C/30 s), annealing (55 °C/30 s), and extension (72 °C/1 min), with a final elongation step at 72 °C for 10 min. PCR products were visualized on a 1.5% agarose gel stained with ethidium bromide to verify the presence of the specific amplicon.

### The antibiotic resistance of *E. coli* isolates

Antibiograms of all identified *E. coli* isolates were determined by the disc diffusion assay according to the guideline of Clinical and Laboratory Standards Institute (CLSI^22^) using Mueller-Hinton agar (Oxoid, Basingstoke, Hampshire, England, UK). Ten antimicrobials related to eight antimicrobial categories were represented in our study, including: Penicillins (Ampicillin, AMP); beta-lactam/Beta-lactamase inhibitor combinations (Amoxicillin-clavulanic acid, AMC); Cephalosporins (Cefotaxime, CTX and Ceftazidime, CAZ); Carbapenems (Imipenem, IPM) ; Aminoglycosides (Gentamicin, CN and Amikacin, AK); Tetracyclines (Tetracycline, TET); Fluoroquinolones (Ciprofloxacin, CIP); and Polymyxins (Colistin, CT). The antimicrobial resistance profiles of the *E. coli* isolates were evaluated using a panel of 10 antibiotics representing 8 distinct pharmacological classes. These agents were selected based on their clinical significance in both human and veterinary medicine, as well as their prevalence of use within the Egyptian poultry sector. The selection included first-line treatments such as ampicillin (Penicillins) and amoxicillin-clavulanic acid (β-lactam/β-lactamase inhibitor combinations), alongside tetracycline and fluoroquinolones (ciprofloxacin), which are historically significant in livestock production. Furthermore, aminoglycosides (gentamicin and amikacin) were included for their efficacy against Gram-negative pathogens. Crucially, the panel incorporated ‘Critically Important Antimicrobials’ according to the One Health framework, including third-generation cephalosporins (cefotaxime and ceftazidime), and last-resort agents for extensively drug-resistant (XDR) infections, namely imipenem (Carbapenems) and colistin (Polymyxins). Monitoring resistance to these specific classes is essential for detecting the spillover of high-priority resistance markers between the poultry environment and human populations.

All drugs were purchased from (Oxoid, England). Escherichia coli American Type Culture Collection (ATCC) 25,922 were used as a reference strain. Isolates were classified into resistance categories based on the standardized international definitions proposed by Magiorakos and his colleagues into; MDR that defined as non-susceptibility to at least one agent in three or more antimicrobial categories, and XDR that defined as non-susceptibility to at least one agent in all but two or fewer antimicrobial categories (i.e., bacterial isolates remain susceptible to only one or two categories)^23^.

Briefly, one colony from the EMB agar plate of each strain was picked up and streaked onto Mueller– Hinton blood agar (Oxoid, UK) and incubated at 37 °C overnight. Bacterial colonies were suspended in 0.9% NaCl and the bacterial concentrations were adjusted using spectrophotometer at 600 nm which equivalent to 1 × 10^8^ colony forming units (CFU)/ml Approximately, 300 µl of the saline suspension was spread onto the surface of a Mueller–Hinton agar plate (Oxoid, UK) using a sterile swab. The antimicrobial discs (Oxoid, UK) of antibiotics were distributed onto the surface of the Mueller–Hinton agar plates using a multi-disc dispenser (Oxoid, UK). The plates were incubated at 37 °C overnight. The diameters of the inhibited zones were measured using sliding callipers and interpreted using standard break points.

### In vitro assessment of the antimicrobial efficacy of AgNPs-H₂O₂ composite

#### Nanocomposite Characterization and Preparation of Working Solutions

The silver nanoparticle-hydrogen peroxide composite (AgNPs–H₂O₂) used in this study was a stabilized commercial formulation (Huwa-San TR-50, obtained from El-Delta Center for Nano Silver Technology, Mansoura, Egypt). According to the manufacturer’s technical specifications, this commercial product is manufactured under strict quality control protocols to ensure high stability and minimal lot-to-lot variability. The morphological attributes of the AgNPs were verified via Transmission Electron Microscopy (TEM), which revealed predominantly spherical particles with an average diameter of 45 nm. This size distribution and morphology are consistent with previously validated characterizations of the same commercial product^24^. According to the manufacturer’s specifications, the stock solution contained 50% hydrogen peroxide integrated with 45 nm silver nanoparticles. To ensure data reproducibility and evaluate potential inter-batch variability, the baseline antimicrobial efficacy (minimum inhibitory concentration; MIC) was cross-verified using different production lots of the formulation, exhibiting consistent biological activity and exceptional stability over the study period.

#### Preparation of working solutions

For the determination of the Minimum Inhibitory Concentration (MIC), a working solution of the AgNPs–H₂O₂ composite was prepared at a concentration of 100 µg/mL, calculated based on the active silver content. A two-fold serial dilution was performed in a 96-well microtiter plate, resulting in a concentration range from 50 µg/mL (Well 1) down to 0.09 µg/mL (Well 10). To ensure a scientifically valid comparison of the comined effect, standalone hydrogen peroxide solutions were prepared at concentrations identical to those present in the corresponding AgNPs–H₂O₂ dilutions. All MIC and MBC values were subsequently calculated and reported based on the active silver concentration.

#### Bacterial consortium preparation and characterization

To evaluate efficacy against a broad spectrum of resistance, a mixed bacterial consortium was prepared. The bacterial consortium was prepared using seven specifically selected *E. coli* isolates (F1-L2, F1-L5, F1-L16, F4-L3, F4-L5, F7-L23, and F8-L22) recovered during Phase I. These strains were chosen based on their high Multiple Antibiotic Resistance (MAR) indices and Extensively Drug-Resistant (XDR) phenotypes, representing the most critical biosecurity threats identified. Detailed information regarding their phenotypic resistance profiles and MAR indices is summarized in Table 1. Selected 7 XDR *E. coli* strains were individually adjusted to 0.5 McFarland standard (1.5 × 10^8^ CFU/mL) in sterile saline. Equal volumes of each suspension were pooled to form a cocktail suspension (homogeneous composite inoculum) ready for use in subsequent assays. The final working inoculum used in antimicrobial assays was adjusted to approximately 1 × 10⁶ CFU/mL.

**Table 1:** Characterization and antimicrobial resistance profiles of the seven XDR *E. coli* strains used for the consortium preparation.

Isolate ID	Source	Phenotypic resistance profile	MAR index	Category
F1-L2	Poultry Litter	AMP, AMC, CTX, CAZ, G, Ak, TET	0.7	XDR
F1-L5	Poultry Litter	AMP, AMC, G, Ak, TET, CIP	0.6	XDR
F1-L16	Poultry Litter	AMP, AMC, CTX, CAZ, IPM, G, Ak, TET, CIP	0.9	XDR
F4-L3	Poultry Litter	AMP, AMC, CTX, IPM, G, Ak, TET	0.7	XDR
F4-L5	Poultry Litter	AMP, AMC, CTX, IPM, Ak, TET	0.6	XDR
F7-L23	Poultry Litter	AMP, AMC, CTX, CAZ, IPM, G, Ak, TET, CIP	0.9	XDR
F8-L22	Poultry Litter	AMP, AMC, CAZ, Ak, TET	0.5	XDR

AMP: Ampicillin; AMC: Amoxicillin-Clavulanic acid; CTX: Cefotaxime; CAZ: Ceftazidime; IPM: Imipenem; G: Gentamicin; Ak: Amikacin; TET: Tetracycline; CIP: Ciprofloxacin.

#### Determination of MIC and MBC

**Fig. 1:**
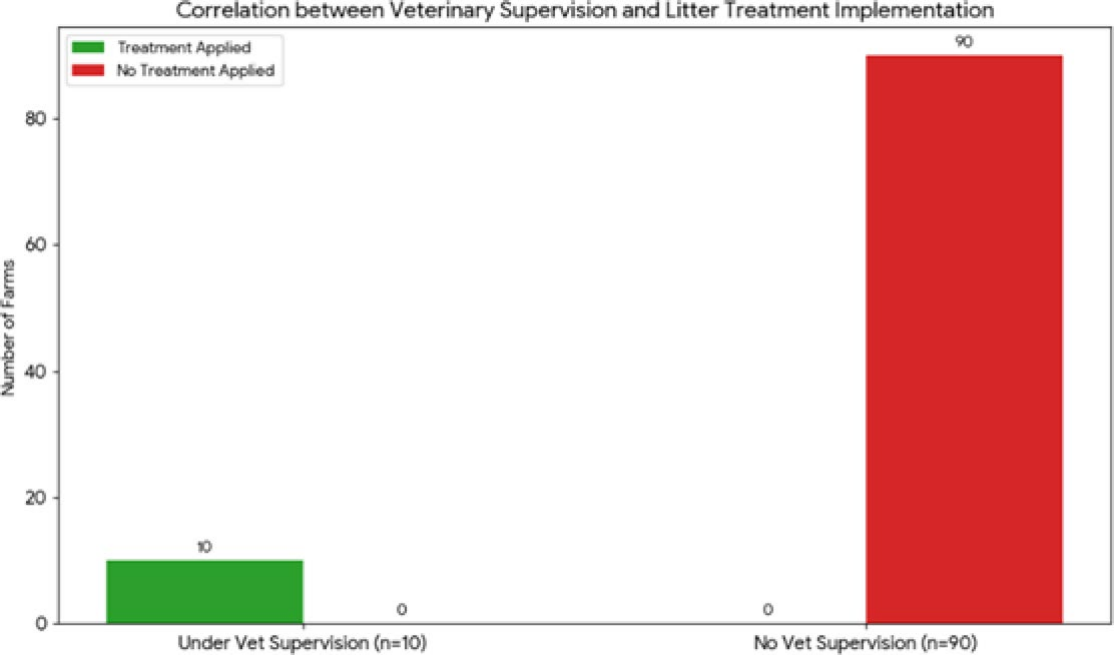
Comparison of litter treatment practices between farms with and without regular veterinary supervision (*N* = 100, *p* < 0.0001). The green bars represent the 10 farms that applied treatment, while the red bar represents the 90 farms that discarded waste without treatment.

**Fig. 2:**
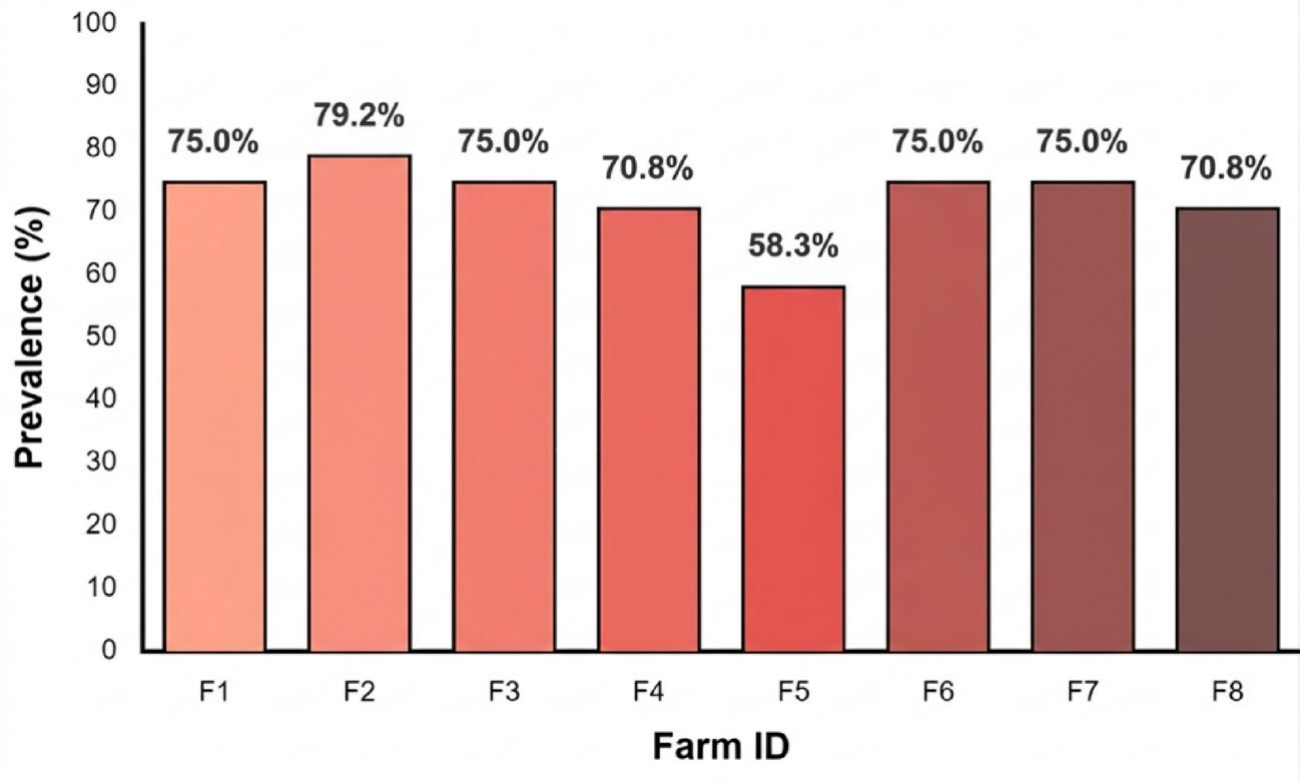
Prevalence of *E. coli* recovered from litter in examined farms (*n* = 8).

**Fig. 3:**
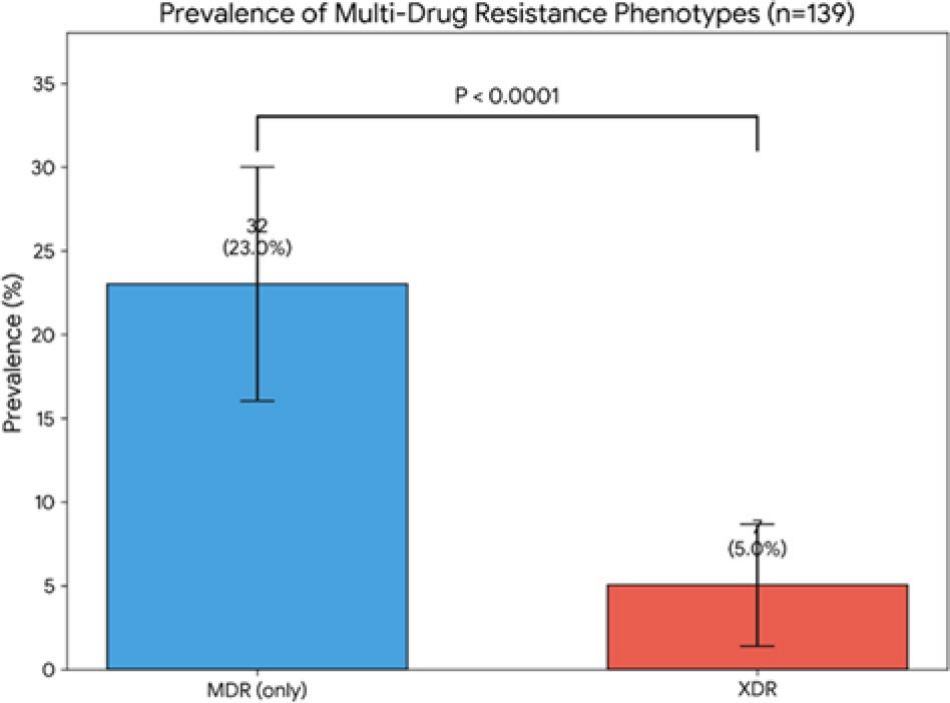
Prevalence of MDR and XDR *E. coli* strains (*n* = 139).

**Fig. 4:**
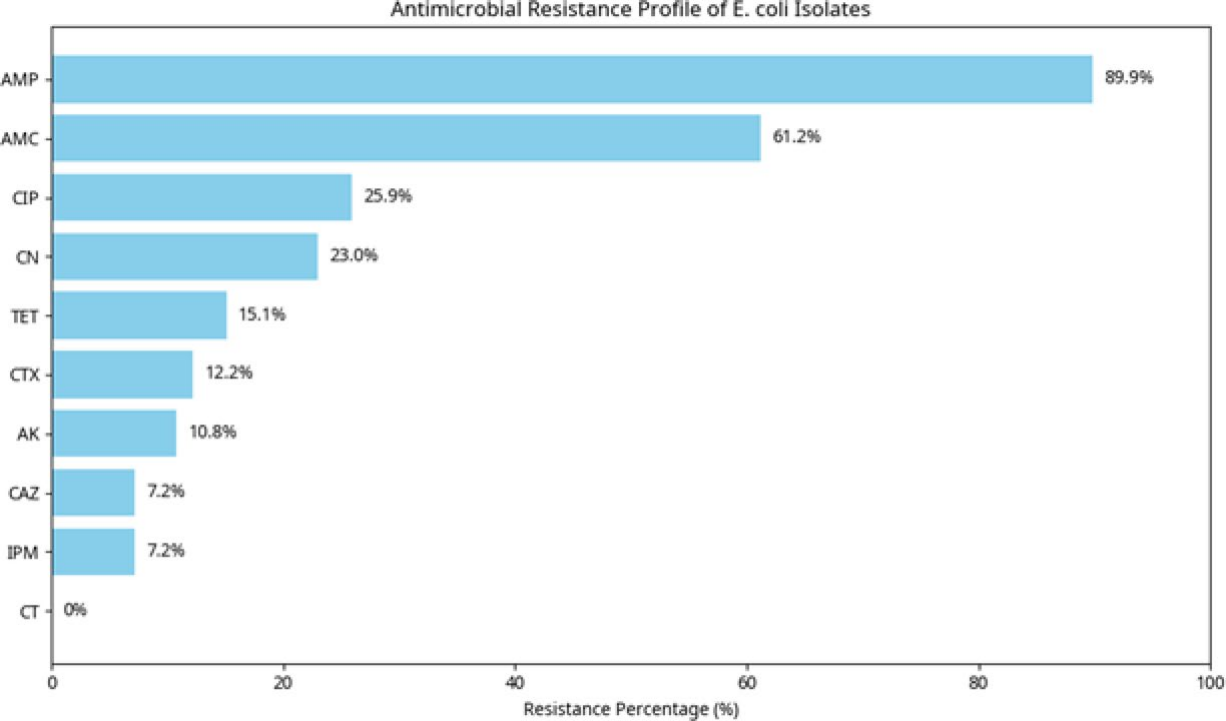
Prevalence of AMR among 139 *E. coli* isolates recovered from poultry litter. The bar chart illustrates the percentage of isolates resistant to each tested antibiotic. AMP: Ampicillin, AMC: Amoxicillin-clavulanic acid, CTX: Cefotaxime, CAZ: Ceftazidime, IPM: Imipenem, CN: Gentamicin, AK: Amikacin, TET: Tetracycline, CIP: Ciprofloxacin, CT: Colistin.

The antimicrobial activity was detected using the broth microdilution method according to CLSI (2018) guidelines (CLSI^25^). Use MIC/MBC (range: 50–0.09 µg/mL). All assays included three biological and three technical replicates. Time-kill kinetics were measured at 0, 1, 6, 12, and 24 h. The procedures were following the previously published protocol using the same commercial formulation^26,24^. Two-fold serial dilutions of the AgNPs-H₂O₂ composite were prepared in Mueller-Hinton Broth (MHB) in sterile 96-well microtiter plates. Each well was inoculated with the bacterial consortium (approx. 1 × 10^6^ CFU/mL). Positive (bacteria without any treatment) and negative (sterility) controls were included. The Minimum Inhibitory Concentration (MIC) was recorded as the lowest concentration exhibiting no visible turbidity after incubation at 37 °C for 24 h. 10 µL aliquots from clear wells were spotted onto nutrient agar to determine the Minimum Bactericidal Concentration (MBC). The MBC was defined as the lowest concentration yielding no colony growth (99.9% killing) after 24 h incubation (Figs. 1, 2, 3,4).

To ensure a scientifically valid comparison, hydrogen peroxide (50%) was used as a control. All working concentrations were freshly prepared prior to experimentation to minimize peroxide decomposition and ensure consistency across experimental groups.

### Time-kill kinetic assay

The bactericidal dynamics were evaluated in sterile 24-well plates as described by^26^. Time-kill assays were performed using each antimicrobial agent at its respective MIC value. The AgNPs–H₂O₂ nanocomposite and hydrogen peroxide (H₂O₂) were tested at concentrations of 3.125 µg/mL and 12.5 µg/mL, respectively. Bacterial suspensions of *E. coli* were exposed to each treatment, and viable counts were determined at predetermined time intervals to evaluate the bactericidal kinetics. A growth control (untreated bacteria) and a hydrogen peroxide (H₂O₂) alone control were included. Aliquots were collected at specific time intervals (0, 1, 6, 12, and 24 h), serially diluted, and plated onto Mueller-Hinton Agar (MHA). Viable counts (CFU/mL) were determined after 24 h of incubation at 37 °C. The results were expressed as Log₁₀ CFU/mL and plotted against time. A reduction of ≥ 3 Log₁₀ CFU/mL relative to the initial inoculum was considered a bactericidal effect. All experiments were performed in triplicate and repeated in three independent experiments.

### Statistical analysis

Initial data entry and management were performed using Microsoft Excel 2016 (Microsoft Corp., Redmond, WA, USA). The normality of continuous data distributions was assessed using the one-sample Kolmogorov–Smirnov test. Data obtained from laboratory results were coded and entered into Statistical Analysis Software (SAS, Version 9.4, SAS Institute, Cary, NC, USA) for analysis. Data collected from the field survey were analyzed using descriptive statistics, including frequencies and percentages to evaluate the survey responses and microbiological data and to describe the distribution of biosecurity gaps and antibiotic usage patterns. The prevalence of *E. coli* and the resistance profiles (MDR/XDR) were expressed as percentages with 95% confidence intervals to ensure the precision of the estimates. A Chi-square test (χ2) was used to assess significance differences in *E. coli* prevalence among the eight studied farms and to compare resistance rates between different antibiotic classes. Resistance variables were coded as binary outcomes (1 = resistant, 0 = sensitive). To account for the multiple comparisons problem and ensure statistical rigor, P-values within the correlation matrix were adjusted using the Benjamini-Hochberg (False Discovery Rate, FDR) procedure. Additionally, point-biserial correlation was used to assess the relationship between the MAR index and individual antibiotic resistance patterns. To visualize the multidrug resistance patterns, a two-way hierarchical clustering heatmap was constructed. The analysis employed Euclidean distance to measure the dissimilarity between isolates and antibiotic classes, while the average linkage method was used to generate the dendrograms. This approach allowed for the simultaneous clustering of farms and antimicrobial agents based on the intensity of resistance profiles. Normality and homogeneity were tested via Shapiro-Wilk and Levene’s tests. Hierarchical clustering used Euclidean distance and average linkage.

**Fig. 5:**
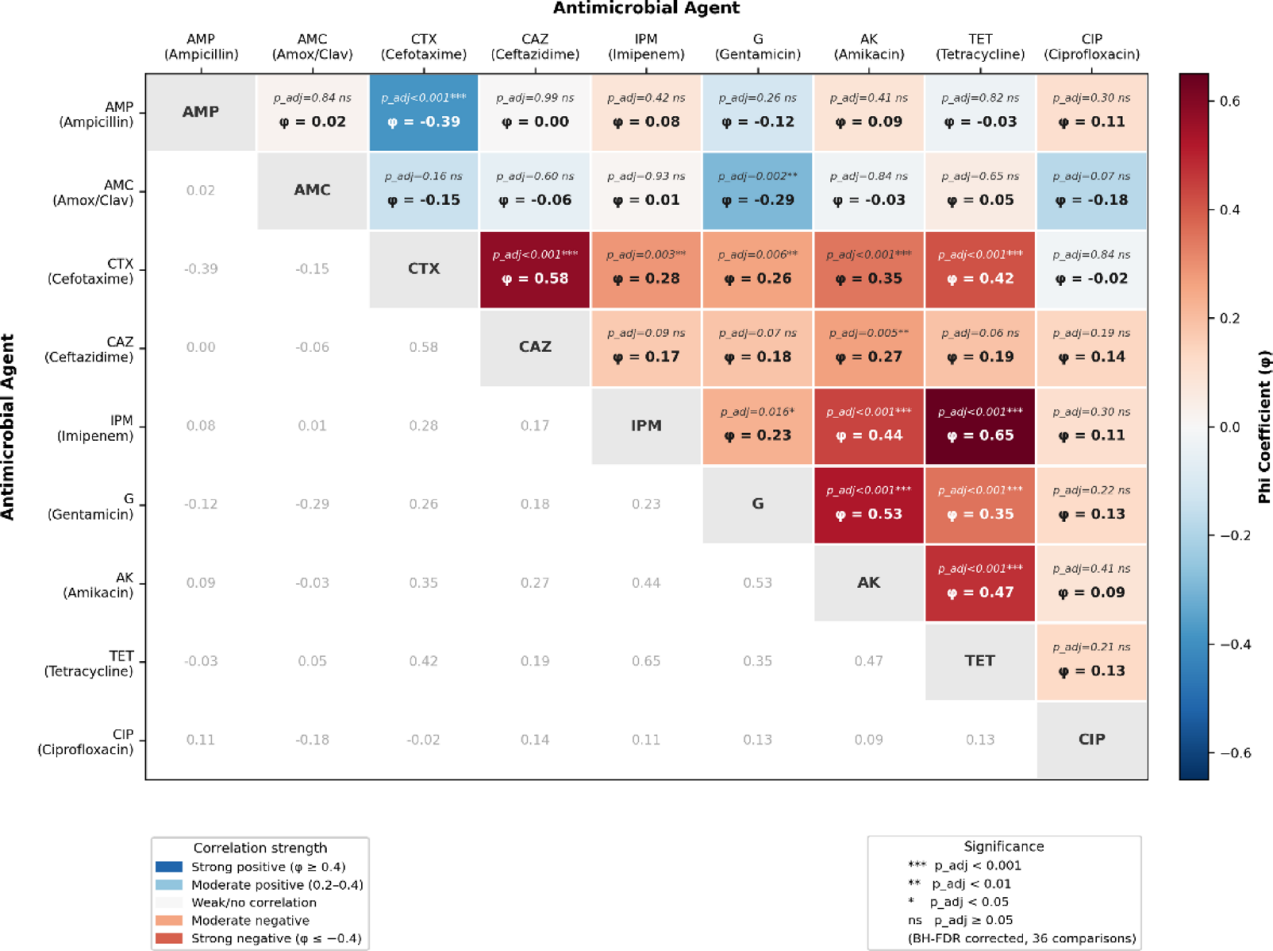
Phi coefficient (φ) correlation matrix displaying pairwise co-resistance associations between antimicrobial agents in *E. coli* isolates (*n* = 139). Color scale ranges from strong negative correlation (dark blue) to strong positive correlation (dark red). The upper triangle shows the phi coefficient (φ) and the BH-FDR adjusted p-value (p_adj) for each pair; the lower triangle shows φ values for reference. Significance levels: *** p_adj < 0.001; ** p_adj < 0.01; * p_adj < 0.05; ns = not significant (p_adj ≥ 0.05). P-values were adjusted for multiple comparisons using the Benjamini-Hochberg False Discovery Rate (BH-FDR) method. AMP: Ampicillin, AMC: Amoxicillin-clavulanic acid, CTX: Cefotaxime, CAZ: Ceftazidime, IPM: Imipenem, CN: Gentamicin, AK: Amikacin, TET: Tetracycline, CIP: Ciprofloxacin.

All in vitro control experiments were performed in triplicate, and the results are expressed as mean ± standard deviation (SD). Statistical significance was evaluated using two-way repeated measures analysis of variance (ANOVA) to assess the effects of treatment, time, and their interaction on bacterial survival (log10 CFU/mL). The normality of data distribution and homogeneity of variances were verified using the Shapiro-Wilk test and Levene’s test, respectively prior to ANOVA. Post-hoc comparisons were performed to identify specific differences between treatment groups at each time point by using Tukey’s Honestly Significant Difference (HSD) test. For all statistical tests, a *P*-value of < 0.05 was considered statistically significant. For the correlation matrix (Fig. 5), phi coefficients (φ) were calculated as the appropriate measure of association for binary resistance phenotypes. To account for multiple comparisons across 36 pairwise tests, all *P*-values were adjusted using the Benjamini-Hochberg False Discovery Rate (BH-FDR) correction (α = 0.05), ensuring that reported associations reflect a controlled rate of false positives. Significance annotations in Fig. 5 are based exclusively on BH-adjusted P-values (p_adj). All statistical analyses and graphical representations were conducted using Python (version 3.11) with SciPy and Matplotlib libraries.

## Results

### Antimicrobial resistance awareness and waste treatment

Regarding the environmental risks associated with poultry waste, the study revealed a significant lack of awareness. A vast majority of the participants (80.0%, 95% CI: 72.2% – 87.8%) were unaware that poultry litter could spread antibiotic-resistant bacteria into the environment. In contrast, only 20.0% (95% CI: 12.2% – 27.8%) verified knowledge of these risks. According to this lack of awareness, the majority of farms (90.0%, 95% CI: 84.1% – 95.9%) reported no pre-disposal treatment (such as composting or chemical disinfection) for the litter. Regarding the barriers to treatment, “Lack of knowledge” and “Lack of time/labor” were the most cited reasons, each representing 40.0% (95% CI: 30.4% – 49.6%) of the responses. (Table 2).

A highly significant association was found between veterinary supervision and the implementation of litter treatment practices (Fisher’s Exact Test, *p* < 0.0001). All farms implementing treatment protocols (10%) were under regular veterinary supervision, while none of the unsupervised farms (90%) performed any form of pre-disposal treatment. (Fig. 1).

**Table 2:** Frequency distribution of poultry litter management practices, biosecurity awareness, and veterinary supervision (*N* = 100).

Variable	Category	Frequency	Total	Percentage (%)	95% confidence interval (CI)
Pre-disposal treatment	No (No composting/disinfection)	90	100	90%	84.1% − 95.9%
	Yes (Biological/Chemical Treatment)	10	100	10%	4.1% − 15.9%
Disposal route	Sold to Fish Farms (Aquaculture)	70	100	70%	61.0% − 79.0%
	Open Land Dumping	20	100	20%	12.2% − 27.8%
	Controlled Landfilling	0	100	0%	0.0% − 3.0%*
	Direct crop fertilizer	10	100	10%	4.1% − 15.9%
	Other methods	0	100	0%	0.0% − 3.0%*
Barriers to treatment	Lack of knowledge	40	100	40%	30.4% − 49.6%
	High cost	20	100	20%	12.2% − 27.8%
	Lack of time/labor	40	100	40%	30.4% − 49.6%
Awareness of AMR	Aware of Environmental AMR spread	80	100	80%	72.2% − 87.8%
	Unaware	20	100	20%	12.2% − 27.8%
Farm disinfection method	Washing with water only	10	100	10%	4.1% − 15.9%
	Traditional (Chlorine/Formalin)	70	100	70%	61.0% − 79.0%
	Advanced disinfectants	20	100	20%	12.2% − 27.8%

These improper disposal practices directly correlate with our laboratory findings, where XDR *E. coli* strains were isolated from the same untreated litter samples, confirming the possibility of pathogen dissemination into the local food chain and aquatic environments.

### Prevalence of *E. coli* in poultry litter

Bacteriological examination of 192 poultry litter samples collected from eight different farms (F1–F8) revealed a high occurrence of *Escherichia coli*. A total of 139 samples were confirmed positive, representing an overall prevalence of 72.40% as shown in Table 3; Fig. 2. The isolation rates varied slightly among the examined farms. Farm F2 recorded the highest prevalence at 79.17% (19/24), meanwhile Farm F5 exhibited the lowest rate at 58.33% (14/24). The statistical analysis showed that there is no significant difference in *E. coli* prevalence among examined farms (Chi square *=* 3.3099, *P* = 0.8549).

**Table 3:** Prevalence of *E. coli* in Poultry Litter from 8 examined farms.

Farm	Positive	Total	Prevalence (%)	95%confidence interval	Chi-Square Statistic and *P*-value
F1	18	24	75.00%	57.68% – 92.32%	Chi square (*χ*^*2*^): 3.3099
F2	19	24	79.17%	62.92% – 95.41%	
F3	18	24	75.00%	57.68% – 92.32%	*P*-value: 0.8549
F4	17	24	70.83%	52.65% – 89.02%	
F5	14	24	58.33%	38.61% – 78.06%	Non-significant
F6	18	24	75.00%	57.68% – 92.32%	
F7	18	24	75.00%	57.68% – 92.32%	
F8	17	24	70.83%	52.65% – 89.02%	
Overall	139	192	72.40%	66.07% – 78.72%	

### Epidemiological dynamics and phenotypic characterization of resistant *E. coli* isolates

Phenotypic AMR profiles, MDR and XDR status, and MAR index of *E. coli* isolates recovered from broiler litter samples (*n* = 139) was detailed in Supplementary Table S1.

#### Prevalence of multidrug-resistant (MDR) and extensively drug-resistant (XDR) strains

The resistance profiles of *E. coli* isolates (*n* = 139) were further categorized into MDR and XDR phenotypes based on their susceptibility patterns revealed from disc diffusion assay. The classification results are summarized in Table 4; Fig. 3. A significant proportion of the isolates, 23.0% (32/139) and 5.0% (7/139) were identified as MDR and XDR strains, respectively at (*P* < 0.0001), highlighting the severity of AMR in the poultry litter environment. The remaining isolates were classified as non-MDR/non-XDR, representing 71.90% (100/139).

**Table 4:** Prevalence of MDR and XDR *E. coli* strains (*n* = 139).

Category	Count	Prevalence (%)	95% Confidence Interval	*P*-value (MDR & XDR)
MDR (only)	32	23.0%	16.0% – 30.0%	< 0.0001*
XDR	7	5.0%	1.4% – 8.7%	
Non-MDR/Sensitive	100	71.90%	64.5% – 79.4%	

#### Phenotypic characterization and ecological risk of AMR

The AMR of 139 *E. coli* isolates was evaluated against ten antimicrobial agents representing different 8 classes. AMP exhibited the highest resistance rate at 89.9% (125/139), followed significantly by AMC at 61.2% (85/139). Moderate resistance was observed against fluoroquinolones, with CIP resistance detected in 25.9% (36/139) of the isolates. Regarding aminoglycosides, CN showed a resistance rate of 23.0%, while AK was more effective with only 10.8% resistance. Unexpectedly, TET resistance was relatively low at 15.1% (21/139), in contrast to characteristic trends in poultry farm environments. Our isolates confirmed high sensitivity to cephalosporins and carbapenems. CTX and CAZ showed low resistance rates of 12.2% and 7.2%, respectively. Also, IPM resistance was negligible at 7.2%. Most markedly, CT was the most effective antimicrobial agent, with 100% susceptibility (0% resistance) (Fig. 4).

### Correlation matrix and co-occurrence patterns of AMR traits

Based on the correlation matrices and the detailed data in Supplementary Table S1& S2. The analysis of co-resistance patterns using the Phi coefficient (*r*_*φ*_) revealed 14 statistically significant pairwise associations after BH-FDR correction (p_adj < 0.05), while 22 pairs were non-significant. The strongest positive association was identified between IPM and TET (φ = 0.65, p_adj < 0.001), suggesting co-selection between carbapenem and tetracycline resistance determinants, likely driven by shared mobile genetic elements. The second strongest was CTX–CAZ (φ = 0.58, p_adj < 0.001), confirming that resistance to one third-generation cephalosporin was highly predictive of resistance to the other, consistent with shared ESBL-mediated mechanisms. Within the aminoglycoside class, G and AK showed a strong positive association (φ = 0.53, p_adj < 0.001), reflecting cross-resistance within the class. Additional highly significant positive associations (p_adj < 0.001) were observed for AK–TET (φ = 0.47), IPM–AK (φ = 0.44), CTX–TET (φ = 0.42), and AMP–CTX in the negative direction (φ = −0.39), the latter indicating that isolates resistant to AMP were significantly less likely to carry cephalosporin resistance, possibly reflecting distinct sub-populations or clonal lineages. Moderately significant associations (p_adj < 0.01) were observed for G–TET (φ = 0.35), CTX–AK (φ = 0.35), AMC–G (φ = −0.29), CTX–IPM (φ = 0.29), and CAZ–AK (φ = 0.27). One additional pair, CTX–G (φ = 0.26), and IPM–G (φ = 0.23, p_adj = 0.016) reached significance at p_adj < 0.05. Notably, several associations that appeared nominally significant before correction — including CIP with AK, AMP, TET, and G, and AMP with AMC — did not survive BH-FDR correction and are reported as non-significant (p_adj ≥ 0.05), underscoring the importance of multiple-comparison adjustment in resistance co-occurrence analyses. All pairwise comparisons were corrected for multiple testing using the Benjamini-Hochberg False Discovery Rate (BH-FDR) method (α = 0.05) across 36 simultaneous comparisons. (Fig. 5). The relationship between resistance indices and specific phenotypes was also measured. The MAR Index showed a strong, highly significant correlation with XDR strains (*r* = 0.73, *p* < 0.001). The MAR Index was also markedly associated with resistance to Amikacin and Tetracycline (*r* = 0.66 for both). These statistical findings are reflected in the phenotypic data, where isolates such as F1, L16 and F7 L23 displayed the highest MAR indices (0.9) due to their extensive resistance profiles.

### Multiple antibiotic resistance (MAR) index (Fig. 6)

**Fig. 6:**
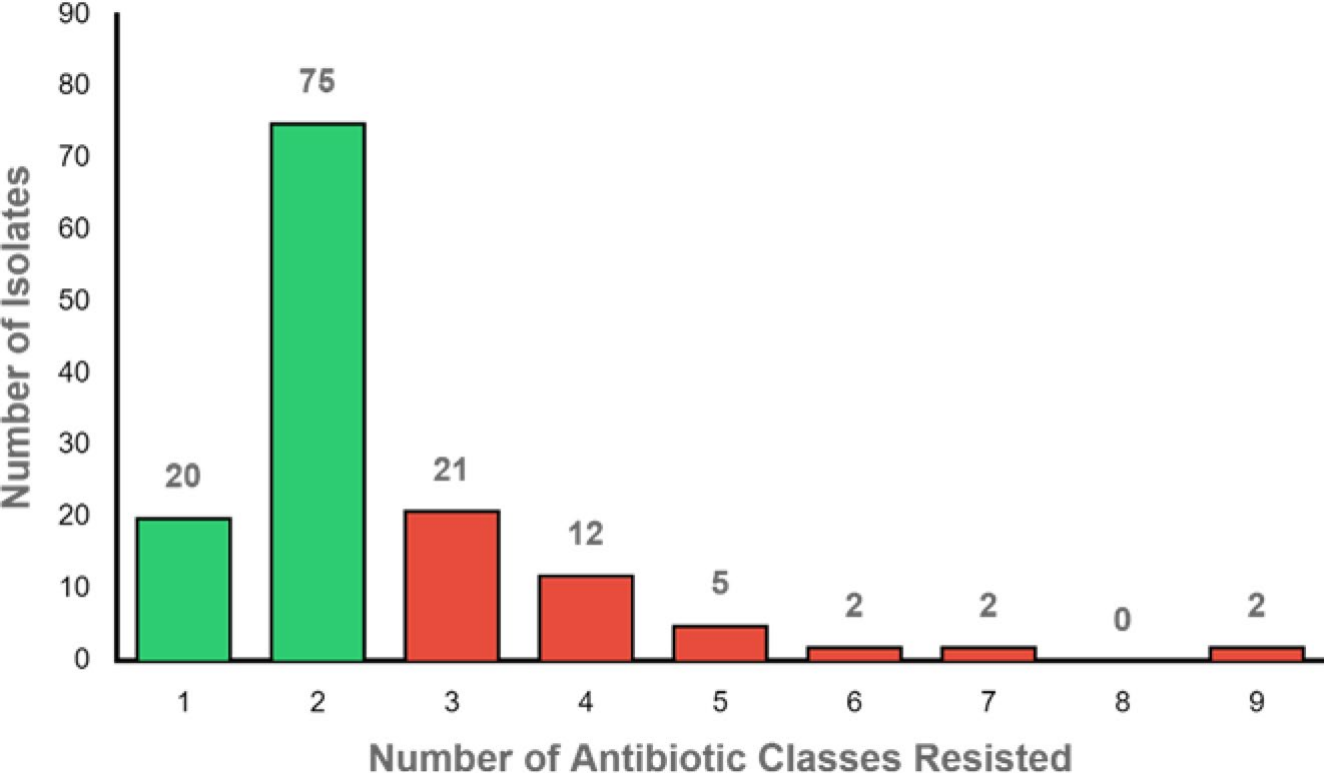
Frequency distribution of multidrug resistance (MDR) profiles among the isolated *E. coli* (*n* = 139). The x-axis represents the number of antibiotic classes to which a single isolate showed resistance. Green bars indicate non-MDR isolates (resistance to < 3 classes), while red bars represent MDR isolates (resistance to 3 classes or more). Numbers above the bars indicate the count of isolates for each resistance category.

The MAR index was calculated for each isolate to evaluate the health risk associated with the spread of resistance in the examined farm environment. As shown in Fig. 6, the MAR index values ranged from 0.0 to 0.9, with an overall average value of 0.24. Analysis of the frequency distribution revealed that 44 isolates (31.7%) had a MAR index greater than 0.2 (> 0.2), while 95 isolates (68.3%) had a MAR index less than or equal to 0.2.

The box plot reveals that a significant proportion of the isolates (31.7%) exhibited MAR indices exceeding the critical threshold of 0.2 (indicated by the red dashed line).

**Fig. 7:**
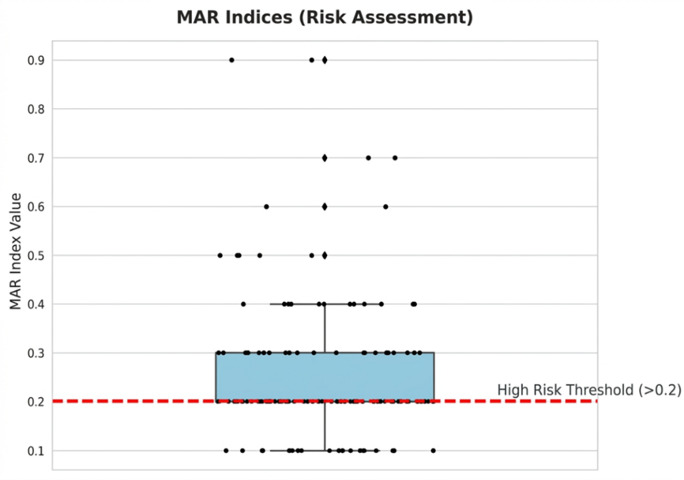
Box plot illustrating the distribution of MAR indices among 139 *E. coli* isolates. The red dashed line at 0.2 represents the critical threshold differentiating low-risk from high-risk sources. Isolates with values above this line are considered to originate from environments with high antibiotic contamination. Black dots represent individual sample distributions. The box represents the interquartile range (IQR), the central horizontal line indicates the median, and whiskers extend to the minimum and maximum values.

### Hierarchical clustering of resistance patterns

**Fig. 8:**
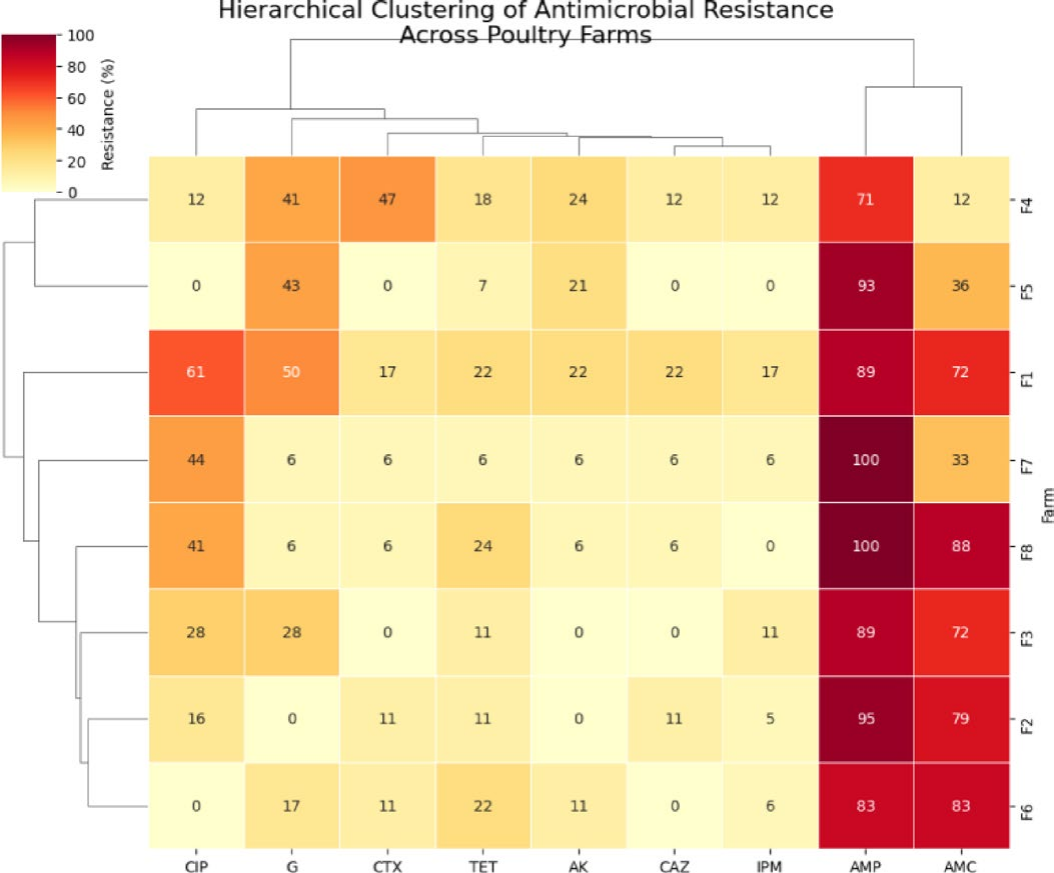
Hierarchical clustering (heatmap) of AMR profiles. The dendrograms (trees) on the left and top represent the clustering of farms and antibiotics, respectively. The color scale ranges from light yellow (low resistance) to dark red (high resistance). Values inside the cells indicate the percentage of resistant isolates. AMP: Ampicillin, AMC: Amoxicillin-clavulanic acid, CTX: Cefotaxime, CAZ: Ceftazidime, IPM: Imipenem, CN: Gentamicin, AK: Amikacin, TET: Tetracycline, CIP: Ciprofloxacin.

Regarding antimicrobial agents, the dendrogram identified two major clusters: a high-resistance cluster comprising Ampicillin and Amoxicillin-clavulanic acid, and a low-resistance cluster grouping antibiotic such as Imipenem, Ceftazidime, and Amikacin. Farm 2 and Farm 8 showed a high resistance cluster, characterized by high resistance rates to multiple drug classes, including tetracyclines and aminoglycosides. In contrast, Farm 5 clustered separately, exhibiting the lowest overall resistance profile among the examined farms (Fig. 8).

### Bactericidal efficacy of AgNPs–H₂O₂ conjugate and time-kill kinetics

#### Minimum inhibitory concentration (MIC) and minimum bactericidal concentration (MBC) analysis

The antimicrobial efficacy of different antimicrobial agents (the AgNPs-H₂O₂ conjugate and H₂O₂ alone) against the XDR *E. coli* Consortium was initially evaluated through measuring MIC and MBC values. As presented in Table 5, the AgNPs-H₂O₂ conjugate demonstrated a significantly lower MIC value of 3.125 µg/mL compared to H₂O₂ alone, which exhibited an MIC of 12.5 µg/mL. Similarly, the MBC values were 6.25 µg/mL and 25 µg/mL for the conjugate and for H₂O₂ alone, respectively. The MBC/MIC ratio for both antimicrobial agents was determined to be 2, indicating a bactericidal mode of action for both agents.

**Table 5:** MIC and MBC values of AgNPs-H₂O₂ conjugate and H₂O₂ alone against XDR *E. coli* Consortium.

XDR E. coli consortium	MIC (µg/mL)	MBC (µg/mL)	MBC/MIC ratio
AgNPs-H₂O₂ conjugate	3.125	6.25	2
H_2_O_2_ alone	12.5	25	2

#### 3.7.2. Kill-time assay: bacterial survival kinetics

**Fig. 9:**
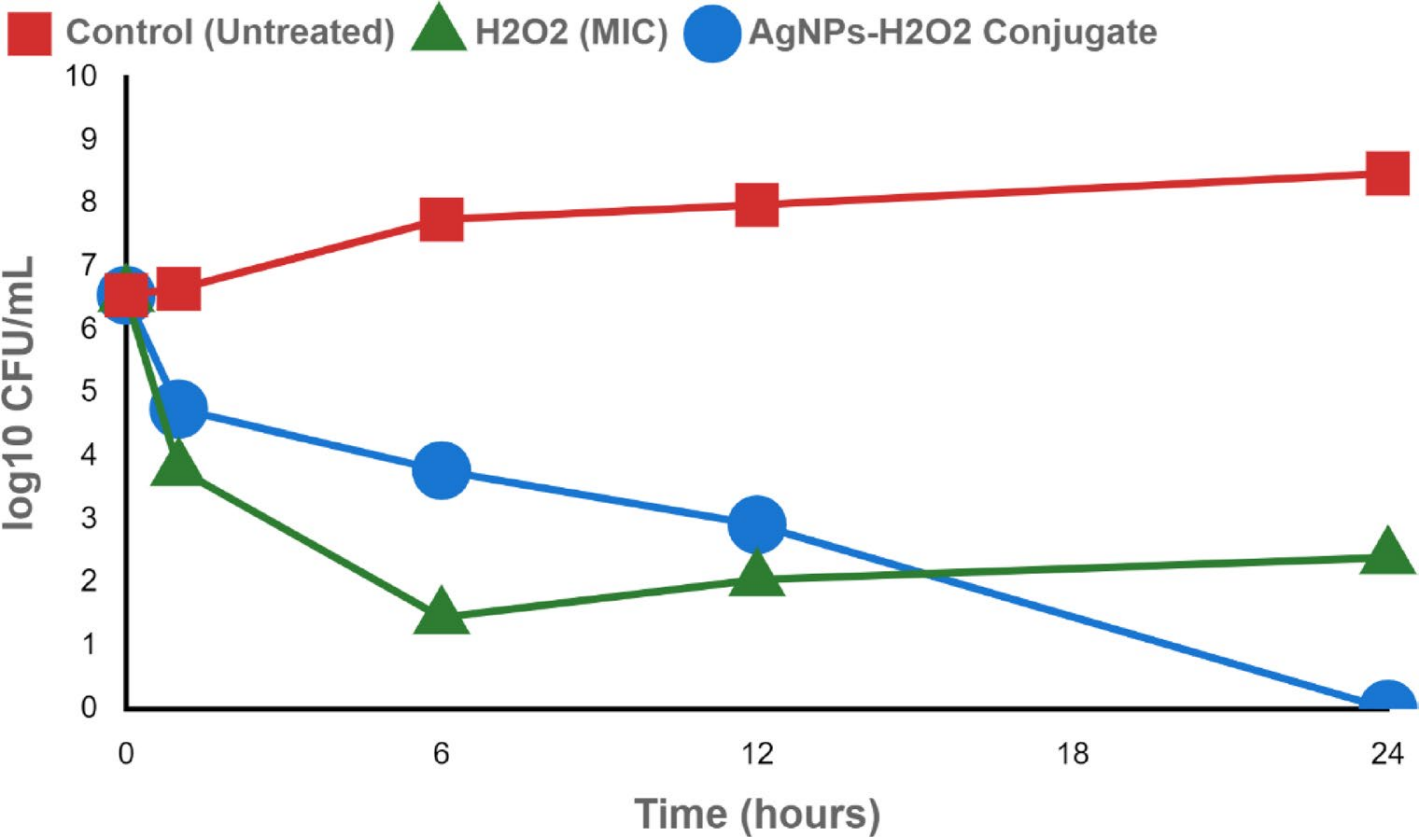
Time-kill kinetics of the AgNPs–H₂O₂ conjugate against XDR *E. coli*. The graph illustrates the bacterial viability over a 24-hour incubation period. The red line represents the untreated control showing typical exponential growth. The green line represents the effect of H₂O₂ alone at its Minimum Inhibitory Concentration (MIC), showing an initial reduction followed by bacterial regrowth. The blue line demonstrates the potent enhacement effect of the AgNPs H₂O₂ conjugate, resulting in sustained bacterial suppression and eventual decline (below the detection limit) at 24 h. Data points represent the mean of three independent experiments (*n* = 3); error bars represent the standard deviation (SD). **P < 0.001 compared to untreated control. .

The Kill time curve was evaluated over a 24-hour period for the two tested antimicrobial agents with results showed in Table 6; Fig. 9. The untreated control group exhibited a constant increase in bacterial population, rising from an initial log_10_ CFU/mL of 6.57 ± 0.07 to 8.47 ± 0.12 after 24 h, revealing normal bacterial growth kinetics.

Treatment with H₂O₂ alone resulted in a rapid initial reduction in bacterial count, decreasing from 6.53 ± 0.06 log_10_ CFU/mL at 0 h to 1.46 ± 0.15 log_10_ CFU/mL at 6 h. However, a subsequent slight increase in bacterial growth was observed, reaching 2.38 ± 0.12 log_10_ CFU/mL by 24 h. This suggests a potential loss of efficacy in the later stages of the assay.

In contrast, the AgNPs-H₂O₂ conjugate demonstrated superior and sustained bactericidal activity. The bacterial count significantly decreased from an initial 6.54 ± 0.02 log_10_ CFU/mL to 4.73 ± 0.10 log_10_ CFU/mL at 1 h, and further to 2.91 ± 0.08 log_10_ CFU/mL at 12 h. Notably, the AgNPs-H₂O₂ conjugate achieved complete reduction of the bacterial growth, with the log_10_ CFU/mL dropping to 0.00 ± 0.00 by 24 h, indicating a potent and long-lasting bactericidal activity.

**Table 6:** Mean and Standard deviation values of Control, AgNPs-H₂O₂ conjugate and H₂O₂ alone against XDR *E. coli* Consortium.

Time (hr)	Control (Mean)	Control (SD)	H_2_O_2__MIC (Mean)	H_2_O_2__MIC (SD)	AgNPs_ H_2_O_2_ (Mean)	AgNPs_ H_2_O_2_ (SD)
0	6.569257681	0.067657862	6.526583641	0.062909357	6.544994517	0.022378066
1	6.636867688	0.064972671	3.782735826	0.081741463	4.733424305	0.097042766
6	7.752527529	0.066509412	1.460070414	0.151237604	3.762038336	0.072033184
12	7.96638642	0.026355402	2.053988872	0.021817236	2.914561776	0.077958931
24	8.471189324	0.119959345	2.379896741	0.124195346	0	0

## Discussion

A key limitation of this study is the restricted access to private poultry farms, which limited microbiological sampling to a subset of farms. This constraint reflects real-world field conditions in Egypt, where farm access is often difficult due to biosecurity concerns and ownership restrictions. Despite this, a relatively large survey sample was achieved, enhancing the contextual relevance of the findings within Dakahlia governorate.

Our study highlights a critical “Knowledge-Practice Gap” in poultry waste management. The high percentage of farmers who do not treat their poultry litter (90%) can be directly related to the high level of unawareness regarding AMR (80%). When farmers are not cognizant of the biological risks — specifically the spread of resistant genes to the environment, they are less likely to invest time or resources into treatment processes like composting or usage of chemical disinfectants. This align with findings documented by^12^.

Furthermore, the fact that 70% of the untreated litter is sold to fish farms poses a severe public health threat. Raw poultry litter in aquatic environments acts as a reservoir for resistant bacteria^13,14^, which can be transferred to humans through the food chain. The study suggests that “High Cost” is not the primary barrier (only 20%); rather, the main obstacles are educational (Lack of knowledge) and operational (Lack of labor). Therefore, intervention strategies should focus on extension services and training programs to educate poultry producers on simple, low-cost composting techniques that can mitigate environmental AMR spread. The statistical significance (*P* < 0.0001) and the visual data in Fig. 1 strongly suggest that veterinary intervention is the most critical factor in mitigating the environmental spread of AMR from poultry waste. Educational efforts should therefore target the 90% of unsupervised farmers to bridge this awareness gap.

The high prevalence of total *E. coli* (72.4%) along with the emergence of critically dangerous extensively drug-resistant (XDR, ~ 5%) and multidrug-resistant (MDR, ~ 23%) sub-populations in our study serves as a severe public health and environmental biosecurity alarm. While molecular characterization of specific antibiotic resistance genes was not performed, these extensive phenotypic profiles reflect a profound accumulation of resistance mechanisms. Crucially, the regional prevalence and persistence of these pathogens directly after bird removal cannot be examined in isolation; rather, they are the direct consequence of a multifactorial interplay between breeding dynamics, management-driven selective pressures, biosecurity thresholds, and regional climatic conditions.

First, the intensive floor-rearing model utilizing deep wood-shaving litter acts as a baseline amplifier for pathogen persistence. Unlike cage systems, continuous floor contact allows for the accumulation of moisture, organic matter, and high fecal shedding, turning the litter into a stable, continuous incubator for enterobacterial proliferation. This microenvironment is further exacerbated by the regional climate of the Dakahlia Governorate (Eastern Nile Delta), where high ambient temperatures and seasonal humidity accelerate organic litter fermentation. The resulting structural heat load compromises the birds’ intestinal integrity, inducing severe physiological stress and triggering enhanced shedding of opportunistic *E. coli* into the environment.

To counteract the biosecurity breaches resulting from sub-optimal ventilation and high stocking densities common in these medium-scale farms, aggressive prophylactic and metaphylactic antibiotic usage is frequently substituted for rigorous hygiene. This constant influx of sub-therapeutic antimicrobial doses exerts an unyielding selective pressure within the warm, moist litter matrix, eliminating susceptible commensals and selectively enriching highly adapted MDR and XDR *E. coli* lineages.

Furthermore, because these farms exhibit lax biosecurity levels (such as inadequate vehicle disinfection, open housing barriers, and the lack of strict rodent/insect control), these enriched resistant clones are easily disseminated between houses and neighboring sectors. This deep ecological interplay explains why our total *E. coli* prevalence, though slightly lower than the 90% in Nigeria^11^ or 86% in India^2^, aligns with the widespread contamination trends documented in developing poultry chains like Cameroon^27^.

Most disturbingly, the verified presence of these resistant isolates immediately after bird removal exposes the complete failure of current biosecurity barriers and traditional cleaning and disinfection (C&D) protocols^9,10^. This environmental persistence strongly aligns with recent genomic findings by Gumede et al., (2026), who identified diverse MDR *E. coli* lineages in poultry environments harboring the *qacI* gene, which confers specific resistance to conventional quaternary ammonium compounds and disinfectants. Such genetic adaptations explain why standard farm sanitation fails and fully justifies the urgent deployment of the advanced AgNPs– H₂O₂ nanocomposite to bypass these specific resistance mechanisms, potentially contributing to interrupting the transmission cycle before its hazardous spillover into aquaculture ecosystems.

In the current study, the average Multiple Antibiotic Resistance (MAR) index was 0.24, with approximately 31.7% (*n* = 44) of the *E. coli* isolates exhibiting a MAR index greater than the critical threshold of 0.2. According to the well-established epidemiological framework by Krumperman^28^, a MAR index exceeding 0.2 is a definitive indicator that the bacterial isolates originate from high-risk environments characterized by intense and continuous selective antibiotic pressure. This high-risk status reflects the extensive and injudicious use of antimicrobials within the surveyed commercial poultry production cycle. Interestingly, the MAR index profiles in our study were relatively lower than those reported by Ibrahim and Habibu (2022), where 100% of the *E. coli* isolates were multidrug-resistant (MDR) with MAR indices ranging from 0.3 to 0.8. This variation could be attributed to differences in regional biosecurity measures, local antimicrobial stewardship, or the specific production management protocols employed across different study areas.

From a One Health perspective, poultry litter represents a volatile reservoir and a critical vector for antimicrobial resistance (AMR) transmission among animals, the environment, and human populations. This is pharmacologically and physiologically supported by Ljubojević et al.^5^, who demonstrated that up to 90% of parent antibiotic compounds administered to poultry are excreted intact in feces, thereby transforming the organic litter matrix into an environmental ‘hot spot’ for horizontal gene transfer (HGT) via mobile genetic elements. The epidemiological risk is further compounded by the persistent nature of these pathogens; as emphasized by Moffo et al.^27^, resistant *E. coli* lineages can survive in stored litter for more than two months. Consequently, standard or inadequate waste disposal practices—such as the direct commercial application of raw poultry litter into aquaculture or agricultural soil—significantly drives the amplification of the environmental MAR index^29^. This critical ecological gap highlights an urgent necessity for implementing advanced, eco-friendly nanocomposite-based disinfection strategies to mitigate the spillover of high-priority resistant pathogens before environmental dissemination.

We observed extremely high resistance to Ampicillin (90%), that consistent with local studies that reported Ampicillin resistance by (100%, 94%, and > 93%)^29,30^, Gumede et al.^31^. Racewicz^32^ attributed this to Class 1 integrons acting as mobile genetic elements. In full agreement, Abuseir and his colleagues confirmed that all APEC strains from broilers in Palestine harbored the intI1 gene alongside blaTEM (100%) and blaSIM (72.3%) genes, underscoring the central role of Class 1 integrons as vectors for beta-lactam and metallo-β-lactamase dissemination in poultry-associated *E. coli*^33^. In contrast, Khong et al., reported a much lower rate (20.6%), highlighting the intense selection pressure exerted by β-lactams in the Egyptian poultry sector compared to other regions^20^. Our study recorded a moderate resistance rate of approximately 25.9% against Fluoroquinolones (Ciprofloxacin). This is remarkably similar to Moffo et al.^27^ that recorded (36%) and significantly higher than the historical 7.55% reported by Talebiyan et al.^34^. However, it remains lower than the alarming 98–99% reported by Ibrahim et al.^30^ and the 70.2% reported by Mandal et al.^2^. in environmental samples. This suggests that while fluoroquinolone resistance is rising, it has not yet reached saturation in the investigated farms. In contrast, research conducted by Gumede et al., (2026) reported 100% resistance against tetracycline, and 76% against trimethoprim–sulfamethoxazole, while ciprofloxacin resistance was rare (3%) as reported by (Gumede et al.^31^). A moderate inverse correlation was observed between Ampicillin and Cefotaxime resistance (*φ *= -0.39). This pattern may reflect differential resistance profiles within the XDR *E. coli* population, suggesting heterogeneous adaptation to β-lactam antibiotics. While the underlying molecular mechanisms were not investigated in this study, the divergent resistance trends indicate that resistance to one β-lactam does not necessarily predict resistance to another within the same class. Such variability in resistance profiles highlights the importance of using broad-spectrum disinfectants with multi-target oxidative activity, such as AgNPs–H₂O₂ nanocomposites, which may reduce the impact of conventional resistance patterns.

A notable divergence in the resistance landscape was observed regarding Colistin, where 100% of the retrieved *E. coli* isolates demonstrated complete phenotypic susceptibility. This complete susceptibility presentation may be partially elucidated by the local antimicrobial management and deployment patterns captured during our initial Phase I field screening. Data obtained from the structured questionnaires across the 100 poultry operations indicated that colistin application was strictly restricted—and practically absent—among the evaluated medium-scale farms that constituted the primary target population for our Phase II intensive sampling.

In contrast to larger-scale, highly industrial integrated operations where prophylactic or growth-promoting colistin usage is occasionally documented in intensive production regions, the lack of active field administration in this specific farm subset profoundly minimized the selective pressure necessary for driving and maintaining phenotypic resistance.

Furthermore, this widespread susceptibility could be closely linked to the distinct physicochemical attributes of the evaluated matrix. Unlike fluid bio-wastes, open aquatic systems, or liquid sewage matrices where plasmid-mediated resistance determinants (such as mobile *mcr* gene families) typically thrive and horizontally disseminate under high moisture dynamics, the relatively dry, alkaline, and high-ammonia microclimates characteristic of the deep wood-shaving poultry litter evaluated in this study likely exert a less permissive environment for the horizontal transmission and long-term retention of metabolic fitness-costly resistance elements in the absence of active drug selection. However, this explanation should be interpreted with caution, as no molecular analysis (e.g., mcr gene detection) was performed, and antimicrobial usage data were based on self-reported practices.

Our results contrast sharply with findings recorded by Hossain et al.^35^ who recorded Colistin susceptibility by (48.84%). Another study from Mandal et al.^2^ detected 15% Colistin resistance in sewage. On other hand, our results are in harmony with another study by Islam et al.^3^ who detected the Colistin susceptibility up to 100% with mcr genes. Colistin as last-resort drug remains effective in our study may be attributed to the hypothesis that damp environments may facilitate the survival of mcr-carrying strains more effectively than the dry litter evaluated in our study. Our results underscore the zoonotic threat and rapid transmission routes of XDR strains between animal species that may be on their way to spread across the food chain, as evidenced by Shafi et al.^36^.

The global escalation of XDR *E. coli* in poultry environments necessitates the development of innovative biocides that can overcome traditional resistance mechanisms. Our results showed decreased MIC by 4-fold from 12.5 µg/mL for H₂O₂ alone to 3.125 µg/mL for the nanocomposite. The MBC/MIC ratio of 2 for both agents confirms their bactericidal nature, meaning they are capable of killing the bacteria rather than merely inhibiting their growth. Our kill time assay study demonstrated the enhanced potency and sustained bactericidal activity of the AgNPs-H₂O₂ conjugate, providing critical insights into its potential as an antimicrobial agent. The untreated control group followed expected growth kinetics. For H₂O₂ alone, a rapid and substantial reduction in bacterial count was observed within the first 6 h, consistent with its bactericidal classification from the MBC/MIC ratio. However, a notable regrowth of the bacterial population occurred between 6 and 24 h. This phenomenon can be attributed to the inherent instability of H₂O₂. Hydrogen peroxide is susceptible to photodegradation and enzymatic breakdown by bacterial catalase, an enzyme commonly produced by bacteria as a defense mechanism against oxidative stress. Consequently, the effective concentration of H₂O₂ in the medium likely diminished over time, allowing a sub-population of surviving bacteria to recover and proliferate.

These initial observations set the stage for understanding the dynamic bactericidal effects observed in the kill-time assay. However, the ability of the nanocomposite to achieve this total bacterial reduction at much lower concentrations (6.25 µg/ml) compared to H₂O₂ (25 µg/ml) is a significant finding for environmental safety. It suggests that terminal disinfection in poultry farms can be achieved with a lower chemical load, thereby reducing environmental toxicity and the selection pressure for further antimicrobial resistance. Our study highlights the potential of the AgNPs–H₂O₂ conjugate as promising approach that may help reduce the transmission of AMR between poultry litter and the surrounding environment by successfully targeting a consortium of XDR isolates.

The exceptional antimicrobial efficacy of the AgNPs–H₂O₂ composite against the XDR *E. coli* consortium can be attributed to a multi-targeted, sequential mechanism operating at the microscopic level. This combined antimicrobial cascade is initiated at the bacterial cell envelope, where the physicochemical properties of the silver nanoparticles govern the primary interaction. Due to their optimized size (~ 45 nm), these nanoparticles exhibit a high surface area-to-volume ratio, enhancing electrostatic interactions with the negatively charged outer membrane of Gram-negative bacteria. Rather than simple adhesion, AgNPs disrupt membrane integrity by inducing structural perturbations and increasing permeability, thereby facilitating the penetration of antimicrobial agents. Similar size-dependent interactions have been reported to enhance nanoparticle uptake in resistant enteric pathogens^24^.

Following membrane destabilization, the mechanism progresses into an intracellular biochemical phase. The compromised membrane permits the influx of H₂O₂ into the cytoplasm, where AgNPs contribute to surface-catalyzed reactive oxygen species (ROS) generation. This interaction accelerates the formation of highly reactive species, including hydroxyl radicals (•OH), leading to extensive oxidative damage to bacterial DNA, proteins, and essential metabolic enzymes^37^. Concurrently, the intracellular release of silver ions (Ag⁺) further disrupts vital cellular processes. By simultaneously targeting multiple cellular components, this combined oxidative and ionic stress effectively circumvents conventional resistance mechanisms, including efflux pumps and enzymatic detoxification pathways^38^, thereby restoring antimicrobial efficacy against extensively drug-resistant strains^39^.

In the present study, the combination of silver nanoparticles and hydrogen peroxide (AgNPs– H₂O_2_) exhibited a profound enhancement in antimicrobial efficacy compared to standalone H₂O₂, as evidenced by the significantly lower MIC and MBC values. It is worth noting that within this commercial formulation (Huwa-San TR-50), silver nanoparticles are integrated at a trace, stabilized concentration. Rather than acting as a primary independent bactericidal agent at high working dilutions, the nano-silver matrix functions predominantly as a catalytic stabilizer. This trace silver prevents the premature, rapid decomposition of hydrogen peroxide and continuously accelerates the generation of highly destructive hydroxyl radicals (• OH) via a Fenton-like reaction. This structural potentiation explains why the combined nanocomposite vastly outperforms standalone H₂O₂. This mechanistic combined antimicrobial effect aligns with the findings of Saad et al.^24^, where the catalytic presence of trace silver was shown to structurally optimize the oxidative stress capacity of hydrogen peroxide against multi-drug resistant pathogens, bypassing the need for higher, potentially toxic concentrations of independent silver nanoparticles.

A critical advantage of this composite lies in its sustained antimicrobial activity. While standalone H₂O₂ is characterized by rapid decomposition and transient efficacy, its association with AgNPs appears to delay degradation and enable a more controlled release profile. This prolonged activity enhances bactericidal efficiency and reduces the likelihood of bacterial regrowth. Such stabilization effects have been similarly observed in advanced nano-enabled disinfectant systems designed to improve antimicrobial persistence against resilient and biofilm-forming pathogens^17^.

From a broader One Health perspective, this nano-composite presents a promising strategy for mitigating antimicrobial resistance dissemination in poultry production systems. A substantial proportion of administered antibiotics is excreted unmetabolized in poultry waste, creating a reservoir for resistant bacteria and horizontal gene transfer^5^. These resistant strains can persist in poultry litter for extended periods^27^, posing environmental and public health risks. The application of the AgNPs–H₂O₂ composite to poultry litter can significantly reduce microbial load while preserving key nutrient components, particularly organic nitrogen. This approach supports the safe recycling of poultry waste into agricultural fertilizers while limiting the environmental spread of resistance determinants. Importantly, this extends to the aquaculture interface, where biogenic selenium nanoparticles have demonstrated efficacy against parasitic infections in Nile tilapia^40^, suggesting that nanoparticle-based interventions represent a broader One Health toolkit capable of interrupting AMR transmission across the animal–environment–food chain continuum. Furthermore, the expanding repertoire of multifunctional biogenic nanoparticles — including selenium NPs synthesized from plant extracts that exhibit combined antiviral, antibacterial, and antioxidant activities^41^— reinforces the principle that nano-enabled disinfection strategies can be tailored for specific ecological niches within complex production systems. Such integrated strategies align with recent efforts to enhance biosecurity and sustainability in livestock production systems^35^.

Despite the outstanding in vitro bactericidal potency and the remarkable reduction in the required chemical concentration achieved by the AgNPs– H₂O_2_ nanocomposite in our laboratory assays, caution must be exercised when extrapolating these findings to real-world agricultural settings. Standard in vitro minimum inhibitory concentration (MIC) determinations are conducted under optimized, clean, and controlled conditions. However, actual poultry production environments and avian bio-wastes present severe practical challenges, most notably high organic matter loads (such as fecal matter and litter debris) and the potential presence of bacterial biofilms. Organic matrices are well-known to interfere with and partially neutralize the oxidative capacity of disinfectants, while biofilms introduce physical barriers that restrict nanoparticle penetration. Therefore, while our preliminary laboratory data underscore the high potential of this nanocomposite as an advanced biosecurity intervention, further field-scale investigations are strictly warranted to fully validate its stability, optimal dosing, and pathogen-reduction efficiency under realistic organic loads and biofilm-associated conditions before wide commercial implementation.

## Conclusions, limitations, and future perspectives

### Conclusions

The findings of this investigation demonstrate a systemic biosecurity deficit across the examined commercial poultry production chain, primarily characterized by a profound deficiency in stakeholder awareness regarding antimicrobial stewardship and an absolute absence of standardized poultry litter treatment protocols. This structural biosecurity failure establishes a high-risk environmental transmission pathway, whereby an alarming prevalence of total *E. coli* along with an emerging sub-population of extensively drug-resistant (XDR) strains is actively channeled into adjacent aquaculture ecosystems through unregulated untreated waste disposal. To mitigate this ecological threat, the synthesized AgNPs– H_2_O_2_ nanocomposite demonstrated highly promising in vitro bactericidal potency, demonstrating strong efficacy against the tested strains within 24 h at a minimum inhibitory concentration (MIC) of 3.125 µg/mL. In laboratory assays, this combined antimicrobial combination allowed for up to a 75% reduction in the required chemical concentration compared to standalone H₂O₂. Consequently, integrating this advanced nanocomposite into routine agricultural hygiene programs presents a potentially valuable component of One Health-based intervention strategies, which may help in interrupting the horizontal transmission cycle of high-priority pathogens from avian bio-waste prior to critical environmental spillover. However, further field-relevant research is strictly required to validate these findings under the influence of real-world organic loads and complex biofilm conditions.

### Study limitations

Despite the promising efficacy and environmental relevance of the developed formulation, several limitations should be acknowledged.

First, regarding the field survey phase, the study was geographically limited to a single region (the Dakahlia Governorate), which may not fully represent the diversity of poultry farming systems and environmental conditions in other areas. Additionally, the data regarding antimicrobial usage patterns were primarily self-reported by the farmers, which potentially introduces recall and social desirability biases, as participants might misremember past treatments or underreport antibiotic use due to perceived social pressure. Due to the lack of formalized, independent veterinary records in some surveyed sectors, independent verification was not fully feasible, although strict confidentiality and on-site visual checks were used to maximize data reliability.

Second, while phenotypic resistance patterns were analyzed, molecular characterization of resistance genes (like mcr gene) and their potential transmission dynamics was not performed.

Third, regarding the nanocomposite evaluation, the antimicrobial assessment was conducted under controlled in vitro conditions; therefore, its effectiveness under field conditions with complex organic loads and bacterial biofilms remains to be validated. Organic matrices are well-known to interfere with and partially neutralize the oxidative capacity of disinfectants, while biofilms introduce physical barriers that restrict nanoparticle penetration, meaning that further field-scale trials are strictly warranted.

Fourth, a comprehensive toxicological and biosafety assessment, particularly regarding the long-term effects on avian health, non-target environmental organisms, and soil microbiota, was beyond the scope of this study.

Addressing these limitations will be essential for confirming the applicability, reliability, and safety of this approach at a larger scale.

### Future perspectives

Future research should focus on validating the performance of this nanocomposite under real field conditions in commercial poultry farms. In vivo studies and environmental safety assessments are needed to ensure its safe application without adverse effects on animals or surrounding ecosystems. Further optimization of the formulation, including improving stability, delivery methods, and cost-effectiveness, would support its potential for large-scale application. In parallel, strengthening veterinary extension programs and promoting evidence-based biosecurity practices could help address the identified gaps in farm management, particularly regarding veterinary supervision and safe waste disposal.

Such integrated efforts may contribute to reducing antimicrobial resistance risks while supporting more sustainable poultry production systems.

## Supplementary Information

Below is the link to the electronic supplementary material.

Supplementary Material 1

## Data Availability

All data supporting the findings of this study are available within the paper and its Supplementary Information. This study was supported by the Deanship of Scientific Research, Vice Presidency forGraduate Studies and Scientific Research, King Faisal University, Saudi Arabia (Grant No.KFU261653).
