# Supplementary material for: Silver nanoparticle hydrogen peroxide composite mitigates resistant Escherichia coli dissemination driven by poultry waste biosecurity failures
Source: Sci Rep. 2026 Jul 16;16:22392. doi: 10.1038/s41598-026-61245-8 (PMC13376196; doi:10.1038/s41598-026-61245-8)
Supplement: Supplementary file 1 — Supplementary Material 1 [file 41598_2026_61245_MOESM1_ESM.docx]

**Table S1. Detailed phenotypic antimicrobial resistance profiles, multidrug resistance (MDR) and extensively drug-resistance (XDR) status, and MAR index of *E. coli* isolates recovered from broiler litter samples.**

| **Positive sample ID** | **phenotypic resistance** | | | | | | | | | | **MDR** | **XDR** | **Mar index** |  |  |  |
| --- | --- | --- | --- | --- | --- | --- | --- | --- | --- | --- | --- | --- | --- | --- | --- | --- |
|  | **AMP** | **AMC** | **CTX** | **CAZ** | **IPM** | **G** | **AK** | **TET** | **CIP** | **COL** |  |  |  |  |  |  |
| F1 L1 | AMP | AMC |  |  |  |  |  |  | CIP | negative | present |  | 0.3 |  | AMP | 125 |
| F1 L2 | AMP | AMC | CTX | CAZ |  | G | AK |  | CIP | negative | present | present | 0.7 |  | AMC | 85 |
| F1 L4 | AMP | AMC |  |  |  | G |  |  | CIP | negative | present |  | 0.4 |  | CTX | 17 |
| F1 L5 | AMP | AMC |  |  |  | G | AK | TET | CIP | negative | present | present | 0.6 |  | CAZ | 10 |
| F1 L6 | AMP | AMC |  |  |  | G | AK |  | CIP | negative | present |  | 0.5 |  | IPM | 10 |
| F1 L8 | AMP |  |  |  | IPM | G |  | TET | CIP | negative | present |  | 0.5 |  | G | 32 |
| F1 L9 | AMP |  |  |  |  |  |  |  |  | negative |  |  | 0.1 |  | AK | 15 |
| F1 L10 | AMP | AMC |  |  |  |  |  |  |  | negative |  |  | 0.2 |  | TET | 21 |
| F1 L12 | AMP |  |  |  |  |  |  |  |  | negative |  |  | 0.1 |  | CIP | 36 |
| F1 L14 | AMP |  |  | CAZ |  | G |  |  | CIP | negative | present |  | 0.4 |  | COL | 0 |
| F1 L16 | AMP | AMC | CTX | CAZ | IPM | G | AK | TET | CIP | negative | present | present | 0.9 |  |  |  |
| F1 L18 |  | AMC |  |  |  |  |  |  | CIP | negative |  |  | 0.2 |  |  |  |
| F1 L19 | AMP | AMC |  |  |  | G |  |  | CIP | negative | present |  | 0.4 |  |  |  |
| F1 L20 | AMP | AMC |  |  |  | G |  |  |  | negative | present |  | 0.3 |  |  |  |
| F1 L21 | AMP |  |  |  | IPM |  |  |  |  | negative |  |  | 0.2 |  |  |  |
| F1 L22 | AMP | AMC | CTX | CAZ |  |  |  |  |  | negative | Present |  | 0.4 |  |  |  |
| F1 L23 | AMP | AMC |  |  |  |  |  |  |  | negative |  |  | 0.2 |  |  |  |
| F1 L24 |  | AMC |  |  |  |  |  | TET | CIP | negative | present |  | 0.3 |  |  |  |
| F2 L1 | AMP |  |  |  |  |  |  |  | CIP | negative |  |  | 0.2 |  |  |  |
| F2 L2 | AMP | AMC |  |  |  |  |  |  | CIP | negative | present |  | 0.3 |  |  |  |
| F2 L3 | AMP |  | CTX | CAZ |  |  |  |  |  | negative |  |  | 0.3 |  |  |  |
| F2 L4 | AMP | AMC |  |  |  |  |  |  |  | negative |  |  | 0.2 |  |  |  |
| F2 L5 | AMP |  | CTX | CAZ |  |  |  |  | CIP | negative | present |  | 0.4 |  |  |  |
| F2 L6 | AMP | AMC |  |  |  |  |  |  |  | negative |  |  | 0.2 |  |  |  |
| F2 L7 | AMP |  |  |  |  |  |  | TET |  | negative |  |  | 0.2 |  |  |  |
| F2 L8 | AMP | AMC |  |  |  |  |  | TET |  | negative | present |  | 0.3 |  |  |  |
| F2 L9 | AMP | AMC |  |  |  |  |  |  |  | negative |  |  | 0.2 |  |  |  |
| F2 L10 | AMP | AMC |  |  |  |  |  |  |  | negative |  |  | 0.2 |  |  |  |
| F2 L11 | AMP | AMC |  |  |  |  |  |  |  | negative |  |  | 0.2 |  |  |  |
| F2 L16 | AMP | AMC |  |  |  |  |  |  |  | negative |  |  | 0.2 |  |  |  |
| F2 L17 | AMP | AMC |  |  |  |  |  |  |  | negative |  |  | 0.2 |  |  |  |
| F2 L18 | AMP | AMC |  |  |  |  |  |  |  | negative |  |  | 0.2 |  |  |  |
| F2 L19 | AMP | AMC |  |  |  |  |  |  |  | negative |  |  | 0.2 |  |  |  |
| F2 L20 | AMP | AMC |  |  |  |  |  |  |  | negative |  |  | 0.2 |  |  |  |
| F2 L22 | AMP | AMC |  |  |  |  |  |  |  | negative |  |  | 0.2 |  |  |  |
| F2 L23 |  | AMC |  |  | IPM |  |  |  |  | negative |  |  | 0.2 |  |  |  |
| F2 L24 | AMP | AMC |  |  |  |  |  |  |  | negative |  |  | 0.2 |  |  |  |
| F3 L2 | AMP | AMC |  |  |  |  |  |  |  | negative |  |  | 0.2 |  |  |  |
| F3 L3 | AMP | AMC |  |  |  |  |  |  |  | negative |  |  | 0.2 |  |  |  |
| F3 L4 | AMP | AMC |  |  |  |  |  |  |  | negative |  |  | 0.2 |  |  |  |
| F3 L6 | AMP | AMC |  |  |  |  |  |  |  | negative |  |  | 0.2 |  |  |  |
| F3 L7 | AMP | AMC |  |  |  |  |  |  |  | negative |  |  | 0.2 |  |  |  |
| F3 L9 |  | AMC |  |  | IPM |  |  |  |  | negative |  |  | 0.2 |  |  |  |
| F3 L10 | AMP | AMC |  |  | IPM |  |  | TET |  | negative | present |  | 0.4 |  |  |  |
| F3 L12 |  | AMC |  |  |  | G |  |  |  | negative |  |  | 0.2 |  |  |  |
| F3 L13 | AMP | AMC |  |  |  |  |  |  |  | negative |  |  | 0.2 |  |  |  |
| F3 L14 | AMP | AMC |  |  |  |  |  |  |  | negative |  |  | 0.2 |  |  |  |
| F3 L16 | AMP | AMC |  |  |  |  |  |  |  | negative |  |  | 0.2 |  |  |  |
| F3 L17 | AMP | AMC |  |  |  | G |  |  |  | negative | present |  | 0.3 |  |  |  |
| F3 L18 | AMP | AMC |  |  |  | G |  | TET |  | negative | present |  | 0.4 |  |  |  |
| F3 L19 | AMP |  |  |  |  | G |  |  | CIP | negative | present |  | 0.3 |  |  |  |
| F3 L21 | AMP |  |  |  |  |  |  |  | CIP | negative |  |  | 0.2 |  |  |  |
| F3 L22 | AMP |  |  |  |  |  |  |  | CIP | negative |  |  | 0.2 |  |  |  |
| F3 L23 | AMP |  |  |  |  | G |  |  | CIP | negative | present |  | 0.3 |  |  |  |
| F3 L24 | AMP |  |  |  |  |  |  |  | CIP | negative |  |  | 0.2 |  |  |  |
| F4 L1 | AMP |  |  |  |  |  |  |  | CIP | negative |  |  | 0.2 |  |  |  |
| F4 L2 | AMP |  |  |  |  |  |  |  | CIP | negative |  |  | 0.2 |  |  |  |
| F4 L3 | AMP | AMC | CTX |  | IPM | G | AK | TET |  | negative | present | present | 0.7 |  |  |  |
| F4 L5 | AMP | AMC | CTX |  | IPM |  | AK | TET |  | negative | present | present | 0.6 |  |  |  |
| F4 L6 |  |  | CTX |  |  |  |  |  |  | negative |  |  | 0.1 |  |  |  |
| F4 L7 |  |  | CTX |  |  | G |  |  |  | negative |  |  | 0.2 |  |  |  |
| F4 L8 |  |  | CTX | CAZ |  |  |  |  |  | negative |  |  | 0.2 |  |  |  |
| F4 L9 |  |  | CTX |  |  |  |  |  |  | negative |  |  | 0.1 |  |  |  |
| F4 L11 | AMP |  |  |  |  |  |  |  |  | negative |  |  | 0.1 |  |  |  |
| F4 L12 | AMP |  |  |  |  |  |  |  |  | negative |  |  | 0.1 |  |  |  |
| F4 L13 | AMP |  |  |  |  | G | AK |  |  | negative |  |  | 0.3 |  |  |  |
| F4 L14 | AMP |  |  |  |  | G |  |  |  | negative |  |  | 0.2 |  |  |  |
| F4 L16 | AMP |  |  |  |  | G |  |  |  | negative |  |  | 0.2 |  |  |  |
| F4 L17 | AMP |  |  |  |  | G | AK |  |  | negative |  |  | 0.3 |  |  |  |
| F4 L19 |  |  | CTX |  |  | G |  |  |  | negative |  |  | 0.2 |  |  |  |
| F4 L21 | AMP |  |  |  |  |  |  |  |  | negative |  |  | 0.1 |  |  |  |
| F4 L22 | AMP |  | CTX | CAZ |  |  |  | TET |  | negative | present |  | 0.4 |  |  |  |
| F5 L2 | AMP | AMC |  |  |  |  |  |  |  | negative |  |  | 0.2 |  |  |  |
| F5 L3 | AMP | AMC |  |  |  |  |  |  |  | negative |  |  | 0.2 |  |  |  |
| F5 L5 |  | AMC |  |  |  |  |  |  |  | negative |  |  | 0.1 |  |  |  |
| F5 L6 | AMP | AMC |  |  |  |  |  |  |  | negative |  |  | 0.2 |  |  |  |
| F5 L9 | AMP |  |  |  |  |  |  |  |  | negative |  |  | 0.1 |  |  |  |
| F5 L10 | AMP |  |  |  |  |  |  |  |  | negative |  |  | 0.1 |  |  |  |
| F5 L12 | AMP | AMC |  |  |  |  | AK | TET |  | negative | present |  | 0.4 |  |  |  |
| F5 L15 | AMP |  |  |  |  | G | AK |  |  | negative |  |  | 0.3 |  |  |  |
| F5 L16 | AMP |  |  |  |  | G | AK |  |  | negative |  |  | 0.3 |  |  |  |
| F5 L17 | AMP |  |  |  |  | G |  |  |  | negative |  |  | 0.2 |  |  |  |
| F5 L18 | AMP |  |  |  |  | G |  |  |  | negative |  |  | 0.2 |  |  |  |
| F5 L19 | AMP |  |  |  |  | G |  |  |  | negative |  |  | 0.2 |  |  |  |
| F5 L20 | AMP |  |  |  |  | G |  |  |  | negative |  |  | 0.2 |  |  |  |
| F5 L21 | AMP |  |  |  |  |  |  |  |  | negative |  |  | 0.1 |  |  |  |
| F6 L1 | AMP |  |  |  |  |  |  |  |  | negative |  |  | 0.1 |  |  |  |
| F6 L2 | AMP |  |  |  | IPM |  |  |  |  | negative |  |  | 0.2 |  |  |  |
| F6 L3 |  |  | CTX |  |  |  | AK | TET |  | negative | present |  | 0.3 |  |  |  |
| F6 L4 | AMP | AMC |  |  |  |  |  |  |  | negative |  |  | 0.2 |  |  |  |
| F6 L5 | AMP | AMC |  |  |  |  |  |  |  | negative |  |  | 0.2 |  |  |  |
| F6 L7 | AMP | AMC |  |  |  |  |  |  |  | negative |  |  | 0.2 |  |  |  |
| F6 L8 | AMP | AMC |  |  |  |  |  |  |  | negative |  |  | 0.2 |  |  |  |
| F6 L9 |  | AMC | CTX |  |  | G |  | TET |  | negative | present |  | 0.4 |  |  |  |
| F6 L10 | AMP | AMC |  |  |  |  |  |  |  | negative |  |  | 0.2 |  |  |  |
| F6 L12 | AMP | AMC |  |  |  |  |  |  |  | negative |  |  | 0.2 |  |  |  |
| F6 L13 |  | AMC |  |  |  |  |  |  |  | negative |  |  | 0.1 |  |  |  |
| F6 L14 | AMP | AMC |  |  |  |  |  |  |  | negative |  |  | 0.2 |  |  |  |
| F6 L15 | AMP | AMC |  |  |  |  |  |  |  | negative |  |  | 0.2 |  |  |  |
| F6 L18 | AMP | AMC |  |  |  |  |  |  |  | negative |  |  | 0.2 |  |  |  |
| F6 L19 | AMP | AMC |  |  |  |  |  |  |  | negative |  |  | 0.2 |  |  |  |
| F6 L20 | AMP | AMC |  |  |  |  |  |  |  | negative |  |  | 0.2 |  |  |  |
| F6 L22 | AMP | AMC |  |  |  | G | AK | TET |  | negative | present |  | 0.5 |  |  |  |
| F6 L24 | AMP | AMC |  |  |  | G |  | TET |  | negative | present |  | 0.4 |  |  |  |
| F7 L1 | AMP |  |  |  |  |  |  |  |  | negative |  |  | 0.1 |  |  |  |
| F7 L2 | AMP |  |  |  |  |  |  |  | CIP | negative |  |  | 0.2 |  |  |  |
| F7 L3 | AMP | AMC |  |  |  |  |  |  |  | negative |  |  | 0.2 |  |  |  |
| F7 L5 | AMP |  |  |  |  |  |  |  |  | negative |  |  | 0.1 |  |  |  |
| F7 L6 | AMP | AMC |  |  |  |  |  |  |  | negative |  |  | 0.2 |  |  |  |
| F7 L7 | AMP |  |  |  |  |  |  |  |  | negative |  |  | 0.1 |  |  |  |
| F7 L9 | AMP |  |  |  |  |  |  |  | CIP | negative |  |  | 0.2 |  |  |  |
| F7 L10 | AMP |  |  |  |  |  |  |  |  | negative |  |  | 0.1 |  |  |  |
| F7 L11 | AMP | AMC |  |  |  |  |  |  |  | negative |  |  | 0.2 |  |  |  |
| F7 L12 | AMP |  |  |  |  |  |  |  | CIP | negative |  |  | 0.2 |  |  |  |
| F7 L14 | AMP |  |  |  |  |  |  |  | CIP | negative |  |  | 0.2 |  |  |  |
| F7 L15 | AMP |  |  |  |  |  |  |  | CIP | negative |  |  | 0.2 |  |  |  |
| F7 L16 | AMP |  |  |  |  |  |  |  | CIP | negative |  |  | 0.2 |  |  |  |
| F7 L20 | AMP |  |  |  |  |  |  |  |  | negative |  |  | 0.1 |  |  |  |
| F7 L21 | AMP | AMC |  |  |  |  |  |  |  | negative |  |  | 0.2 |  |  |  |
| F7 L22 | AMP |  |  |  |  |  |  |  |  | negative |  |  | 0.1 |  |  |  |
| F7 L23 | AMP | AMC | CTX | CAZ | IPM | G | AK | TET | CIP | negative | present | present | 0.9 |  |  |  |
| F7 L24 | AMP | AMC |  |  |  |  |  |  | CIP | negative | present |  | 0.3 |  |  |  |
| F8 L3 | AMP | AMC |  |  |  |  |  |  | CIP | negative | present |  | 0.3 |  |  |  |
| F8 L4 | AMP | AMC |  |  |  |  |  |  | CIP | negative | present |  | 0.3 |  |  |  |
| F8 L5 | AMP | AMC |  |  |  |  |  |  | CIP | negative | present |  | 0.3 |  |  |  |
| F8 L6 | AMP | AMC |  |  |  |  |  |  | CIP | negative | present |  | 0.3 |  |  |  |
| F8 L7 | AMP | AMC |  |  |  |  |  |  |  | negative |  |  | 0.2 |  |  |  |
| F8 L8 | AMP | AMC |  |  |  |  |  |  |  | negative |  |  | 0.2 |  |  |  |
| F8 L9 | AMP | AMC |  |  |  |  |  |  |  | negative |  |  | 0.2 |  |  |  |
| F8 L10 | AMP | AMC |  |  |  |  |  |  |  | negative |  |  | 0.2 |  |  |  |
| F8 L11 | AMP | AMC |  |  |  | G |  | TET |  | negative | present |  | 0.4 |  |  |  |
| F8 L12 | AMP | AMC |  |  |  |  |  |  |  | negative |  |  | 0.2 |  |  |  |
| F8 L13 | AMP | AMC |  |  |  |  | AK | TET | CIP | negative | present |  | 0.5 |  |  |  |
| F8 L14 | AMP | AMC |  |  |  |  |  |  | CIP | negative | present |  | 0.3 |  |  |  |
| F8 L15 | AMP | AMC |  |  |  |  |  |  |  | negative |  |  | 0.2 |  |  |  |
| F8 L18 | AMP | AMC |  |  |  |  |  | TET |  | negative | present |  | 0.3 |  |  |  |
| F8 L19 | AMP |  |  |  |  |  |  |  |  | negative |  |  | 0.1 |  |  |  |
| F8 L21 | AMP |  | CTX |  |  |  |  |  |  | negative |  |  | 0.2 |  |  |  |
| F8 L22 | AMP | AMC |  | CAZ |  |  |  | TET | CIP | negative | present | present | 0.5 |  |  |  |

**Supplementary Table S2.** **Pairwise Correlation matrix showing Phi coefficient (𝜙) values and Benjamini–Hochberg adjusted statistical significance (p-values) for all pairs of tested antimicrobial agents based on the phenotypic profiles in Table S1**

| **Antimicrobial Agent** | **AMP** | **AMC** | **CTX** | **CAZ** | **IPM** | **G** | **AK** | **TET** | **CIP** |
| --- | --- | --- | --- | --- | --- | --- | --- | --- | --- |
| **AMP (Ampicillin)** | 1 |  |  |  |  |  |  |  |  |
| **AMC (Amox/Clav)** | 0.02 | 1 |  |  |  |  |  |  |  |
| **CTX (Cefotaxime)** | -0.39*** | -0.15 | 1 |  |  |  |  |  |  |
| **CAZ (Ceftazidime)** | 0 | -0.06 | 0.58*** | 1 |  |  |  |  |  |
| **IPM (Imipenem)** | 0.08 | 0.01 | 0.28** | 0.17 | 1 |  |  |  |  |
| **G (Gentamicin)** | -0.12 | -0.29** | 0.26** | 0.18 | 0.23* | 1 |  |  |  |
| **AK (Amikacin)** | 0.09 | -0.03 | 0.35*** | 0.27** | 0.44*** | 0.53*** | 1 |  |  |
| **TET (Tetracycline)** | -0.03 | 0.05 | 0.42*** | 0.19 | 0.65*** | 0.35*** | 0.47*** | 1 |  |
| **CIP (Ciprofloxacin)** | 0.11 | -0.18 | -0.02 | 0.14 | 0.11 | 0.13 | 0.09 | 0.13 | 1 |

Values represent the Phi Coefficient (phi) for binary phenotypic resistance associations. Asterisks denote statistically significant correlations after rigorous formal adjustment for multiple comparisons using the Benjamini–Hochberg False Discovery Rate (BH-FDR) method across distinct pairwise comparisons:

- *P< 0.05 (Significant)
- **P< 0.01 (Highly Significant)
- ***P< 0.001 (Extremely Significant)
- Absence of an asterisk indicate non-significant relationship

### Appendix S1: Survey Questionnaire for Poultry Waste Management and Biosecurity Assessment

### ملحق 1: استبيان مسح إدارة مخلفات الدواجن وتقييم الأمن الحيوي

#### General Information / معلومات عامة:

- **Farm Code (ID) / رمز المزرعة:** ....................
- **Date / التاريخ:** ..../..../……
- **Location (Sub-district/Village) / الموقع (المركز/القرية):** ....................

#### Part 1: Farm Eligibility & Production Characteristics (Phase I Screening)

#### الجزء الأول: أهلية المزرعة وخصائص الإنتاج (تصفية المرحلة الأولى)

*This section determines the enrollment of the farm into the intensive Phase II bio-sampling cohort based on predefined criteria.* *يحدد هذا القسم مدى أهلية المزرعة للانضمام إلى مجموعة أخذ العينات المكثفة للمرحلة الثانية بناءً على معايير مسبقة.*

**1. What is the production capacity of the farm per cycle?** **ما هي السعة الإنتاجية للمزرعة في الدورة الواحدة؟**

- [ ] Less than 5,000 birds / أقل من 5,000 طائر
- [ ] 5,000 to 10,000 birds / من 5,000 إلى 10,000 طائر
- [ ] More than 10,000 birds / أكثر من 10,000 طائر

**2. What type of rearing system and litter matrix is employed?** **ما هو نظام التربية ونوع الفرشة المستخدمة في المزرعة؟**

- [ ] Intensive floor rearing with deep wood-shaving litter / تربية أرضية مكثفة مع فرشة نشارة خشب عميقة
- [ ] Cage rearing system (no litter) / نظام تربية في بطاريات (بدون فرشة)
- [ ] Other system / نظام آخر: ....................

**3. Does the farm have a documented history of antibiotic usage during the rearing cycle?** **هل تمتلك المزرعة تاريخاً موثقاً لاستخدام المضادات الحيوية خلال دورة التربية؟**

- [ ] Yes (intensive/routine use for prophylaxis or treatment) / نعم (استخدام مكثف/روتيني للوقاية أو العلاج)
- [ ] No (rare or no antibiotic usage) / لا (استخدام نادر أو منعدم للمضادات الحيوية)

#### Part 2: Litter Management and One Health Interface

#### الجزء الثاني: إدارة الفرشة ورابط الصحة المشتركة

4. How do you commercially dispose of the poultry litter after the depopulation phase? **كيف تتخلص تجارياً من فرشة الدواجن (السبلة) بعد إخلاء العنبر؟**

- [ ] Selling/Giving it directly to fish farms (aquaculture) / بيعها أو إعطاؤها مباشرة لمزارع الأسماك
- [ ] Selling/Using it directly as crop fertilizer / بيعها أو استخدامها مباشرة كسماد للمحاصيل
- [ ] Disposal in open areas or public waterways near the farm / الرمي في مناطق مفتوحة أو مجاري مائية قريبة
- [ ] Other practices / ممارسات أخرى: ....................

5. Do you apply any formal treatment (e.g., composting, chemical disinfection, sun-drying) to the litter before it leaves the farm? **هل تقوم بأي نوع من المعالجة الفعلية (مثل الكومبوست، التطهير الكيميائي، التجفيف الشمسي) للفرشة قبل خروجها من المزرعة؟**

- [ ] Yes / نعم
- [ ] No / لا

6. If "No", what is the primary reason for not treating the poultry litter? **إذا كانت الإجابة "لا"، ما هو السبب الرئيسي لعدم معالجة الفرشة؟**

- [ ] Lack of technical knowledge regarding proper methods / نقص المعرفة التقنية بالطرق الصحيحة
- [ ] High economic cost of treatment operations / التكلفة الاقتصادية العالية لعمليات المعالجة
- [ ] Lack of available time or specialized labor / نقص الوقت المتاح أو العمالة المتخصصة
- [ ] No perceived biological or environmental risk / عدم الشعور بوجود مخاطر بيولوجية أو بيئية للمخلفات

#### Part 3: Biosecurity Awareness and Post-Depopulation Sanitation

#### الجزء الثالث: الوعي بالأمن الحيوي والتطهير بعد إخلاء العنبر

7. Are you aware that untreated poultry waste can disseminate multidrug-resistant (MDR/XDR) bacteria into surrounding aquatic and agricultural environments? **هل تعلم أن مخلفات الدواجن غير المعالجة يمكن أن تنشر بكتيريا مقاومة متعددة للأدوية في البيئات المائية والزراعية المحيطة؟**

- [ ] Yes / نعم
- [ ] No / لا

8. How do you typically clean and disinfect the farm environment after complete litter removal? **كيف تقوم عادةً بتنظيف وتطهير مباني المزرعة بعد إزالة الفرشة بالكامل؟**

- [ ] Washing with water only (no chemical disinfectants) / الغسيل بالماء فقط (بدون مطهرات كيميائية)
- [ ] Using traditional chemical disinfectants (e.g., Chlorine, Formalin) / استخدام مطهرات كيميائية تقليدية (مثل الكلور، الفورمالين)
- [ ] Using advanced multi-component disinfectants / استخدام مطهرات متطورة متعددة المكونات
- [ ] No post-depopulation disinfection practiced / لا يتم إجراء أي تطهير بعد إخلاء العنبر
